# Syrphid Diversity in Sweet Alyssum Flower Strips in Quebec's Lettuce Fields: Molecular Identification and Delimitation of the *Sphaerophoria* Complex

**DOI:** 10.1002/ece3.72145

**Published:** 2025-09-11

**Authors:** Malek Kalboussi, Alice Dabrowski, Andrew D. Young, Annie‐Ève Gagnon, Colin Favret

**Affiliations:** ^1^ Département de sciences biologiques, Institut de recherche en biologie végétale Université de Montréal Montréal Quebec Canada; ^2^ Saint‐Jean‐sur‐Richelieu Research and Development Centre Agriculture and Agri‐Food Canada Saint‐Jean‐sur‐Richelieu Quebec Canada; ^3^ School of Environmental Sciences University of Guelph Guelph Ontario Canada

**Keywords:** aphidophagous, conservation biological control, hover flies, *Lactuca sativa*, species complex, *Sphaerophoria*, *Toxomerus marginatus*

## Abstract

Lettuce is an economically significant crop in Canada, with 70% of commercial production occurring in peatlands (Histosols) in southern Quebec. Insecticide application is currently the main method for managing lettuce pests, but there is a growing need for sustainable pest control alternatives. Conservation biological control, such as incorporating flowering strips into fields to attract natural enemies, is a promising strategy. This study evaluates the potential of sweet alyssum, *Lobularia maritima* , to attract syrphids, whose larvae are voracious predators of lettuce pests, particularly aphids. A total of 16 species were collected from flowering plants across three lettuce farms in Quebec. The most abundant species was *Toxomerus marginatus* , accounting for 70% of all specimens, followed by *Sphaerophoria philanthus* (10.3%) and *Allograpta obliqua* (4.6%). All other species each constituted less than 4% of the total catch. A subset of 82 females from the *Sphaerophoria philanthus/asymmetrica/abbreviata* species complex underwent COI DNA‐based delimitation analyses, revealing three molecular operational taxonomic units (MOTUs). Fourteen of the 16 identified species or MOTUs are aphidophagous. Analysis of diversity metrics across the three sites indicated no statistically significant differences between flower and control treatments. However, of the 16 species recorded, 10 were found exclusively or predominantly (≥ 80%) in flower plots. Our findings suggest that alyssum flowers can successfully attract natural aphid predators in lettuce fields. This approach has the potential to mitigate lettuce pest issues and reduce reliance on insecticides, thus promoting more sustainable pest management.

## Graphical abstract

This study demonstrates that sweet alyssum effectively attracts syrphid species, the majority of which are aphid predators, making it a promising tool for biological control in Quebec lettuce fields. With *Toxomerus marginatus* as the most abundant species, the research also underscores taxonomic challenges within the *Sphaerophoria* complex and provides a DNA barcode reference to aid in species identification.

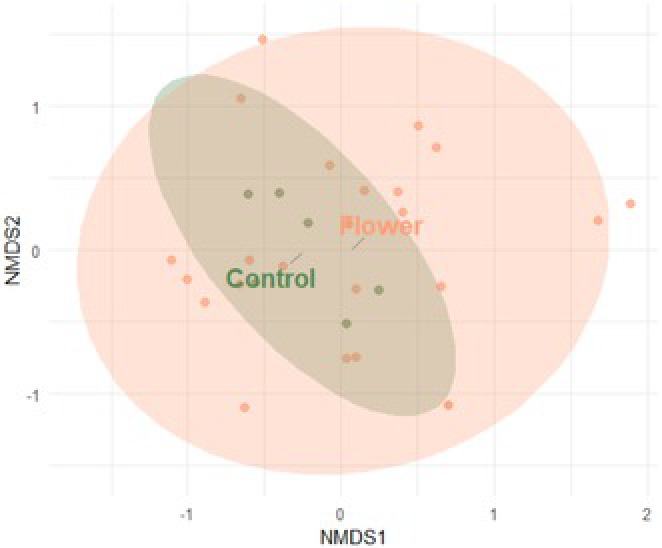

## 1 Introduction

Lettuce ( *Lactuca sativa* L.), an annual vegetable from the Asteraceae family, is a key agricultural product in Canada. In 2023, 3851 ha were cultivated with a market value of approximately 100 million CAD (Government of Canada 2023). Quebec leads lettuce production in the country, contributing 87% of field‐grown lettuce and 63% of greenhouse‐grown lettuce (AAFC 2021). Notably, 70% of Canada's lettuce is grown on organic peatlands (Histosols) in southern Quebec (AAFC 2021).

Lettuce is vulnerable to many insect pests, with aphids (Hemiptera: Aphididae) being among the most economically damaging (Stufkens and Teulon 2003; Holman 2008). In Canada, the key aphid species affecting lettuce are *Acyrthosiphon lactucae* (Passerini), *Aulacorthum solani* (Kaltenbach), *Macrosiphum euphorbiae* (Thomas), *Myzus persicae* (Sulzer), and *Nasonovia ribisnigri* (Mosley) (Díaz et al. 2007; Matthews 2017), with *N. ribisnigri* being the most economically important pest in Quebec lettuce (AAFC 2021). These lettuce aphids colonize all parts of the plant except the roots (Díaz et al. 2007). Their infestations may cause minor stunting and honeydew deposits, but they are not significant virus vectors (Musa et al. 2020). However, the presence of live aphids inside the lettuce head often renders the crop unmarketable, leading to serious economic consequences (Matthews 2017; Niemann and Poehling 2022).

The primary method for managing lettuce pests in Canada relies on synthetic pesticides (Malaj et al. 2020). In Quebec, lettuce fields typically receive two to three insecticide treatments per season, with pesticide residues found on over 63% of lettuce samples harvested from grocery store shelves (MAPAQ 2020). Considering the detrimental effects of pesticides on human health and the environment (Klarich et al. 2017; Montiel‐León et al. 2019), alternative pest management strategies are being explored. Lettuce aphids pose a particular challenge due to their hidden location in heart leaves, making them difficult to control with conventional methods (Rufingier et al. 1997; Niemann and Poehling 2022).

Conservation biological control, the promotion of pests' natural enemies through habitat management, offers a promising alternative to pesticide use (Eilenberg et al. 2001). For example, rolled‐rye cover crops in lettuce fields have been effective at supporting predator and parasitoid populations and thereby reducing *N. ribisnigri* colonization (Dumotier et al. 2024). In addition, flower strips in proximity to the primary crop can attract natural enemies of crop pests by providing essential resources such as nectar, pollen, shelter, and alternative prey (Colley and Luna 2000; Morandin et al. 2014; Tschumi, Albrecht, Bärtschi, et al. 2016; Tschumi, Albrecht, Collatz, et al. 2016; Albrecht et al. 2020; Hogg et al. 2023; Zhong et al. 2024). This method of conservation biological control has been studied and validated across different agricultural systems, showing promising results in increasing the diversity of beneficial insects and enhancing ecosystem services, such as pollination and pest control (Brennan 2013, 2016; Badenes‐Pérez 2019; Kordbacheh et al. 2020; Mateos‐Fierro et al. 2021; Fountain 2022; Köneke et al. 2023; Scarlato et al. 2023; Zhong et al. 2024).

Syrphids exhibit different dietary regimes, functioning as both pollinators and predators (Dunn et al. 2020). Syrphid larvae are common predators of aphids (Hickman and Wratten 1996), with the most voracious species consuming more than 160 lettuce aphids daily (Hopper et al. 2011). Attracted to floral resources for pollen and nectar, adult syrphids are particularly responsive to the presence of flower strips (Landis et al. 2000; Irvin et al. 2021). Sweet alyssum, *Lobularia maritima* (L.) Desv (Brassicaceae), has proven especially effective in enhancing syrphid populations in vegetable crops, outperforming other species of flowers (Ambrosino et al. 2006; Hogg et al. 2011; Haris‐Cypher et al. 2023; Killewald et al. 2024; Zhong et al. 2024). Research conducted in California has specifically highlighted sweet alyssum's capacity to support syrphid populations in lettuce crops (Chaney 1998; Chaney 2003; Smith and Chaney 2007; Bugg et al. 2008; Smith et al. 2008; Gillespie et al. 2011). Furthermore, sweet alyssum flowers throughout the growing season (Picó and Retana 2003; Brennan 2016), providing a consistent supply of nectar and pollen. Its flower's short corolla and large breadth make for easy accessibility to syrphids, which have short mouthparts (Campbell et al. 2012; Ribeiro and Gontijo 2017).

Most research on flower strips in lettuce production has primarily focused on their effects on crop quality and yield (Brennan 2013), economic aspects (Martinez et al. 2024), and land use efficiency (Martinez et al. 2024). Relatively few studies, mainly limited to the western part of North America, have explored the impact of flower strips on enhancing populations of natural enemies of lettuce pests (Pascual‐Villalobos et al. 2006; Smith and Chaney 2007; Smith et al. 2008; Hogg et al. 2023). Understanding which syrphid species can be attracted by sweet alyssum is essential for promoting effective biological control of aphids in eastern North American lettuce production. This study aimed to fill knowledge gaps by examining the richness of syrphids in sweet alyssum flower strips adjacent to lettuce crops in the Histosols of southern Quebec. Using both morphological and molecular methods, we explored the diversity of syrphid populations and established a DNA barcode reference library of syrphid species collected in sweet alyssum. Our checklist and database will aid in the identification of both adult and larval syrphids.

## 2 Materials and Methods

### 2.1 Field Site and the Establishment of Flower Strips

**FIGURE 1:**
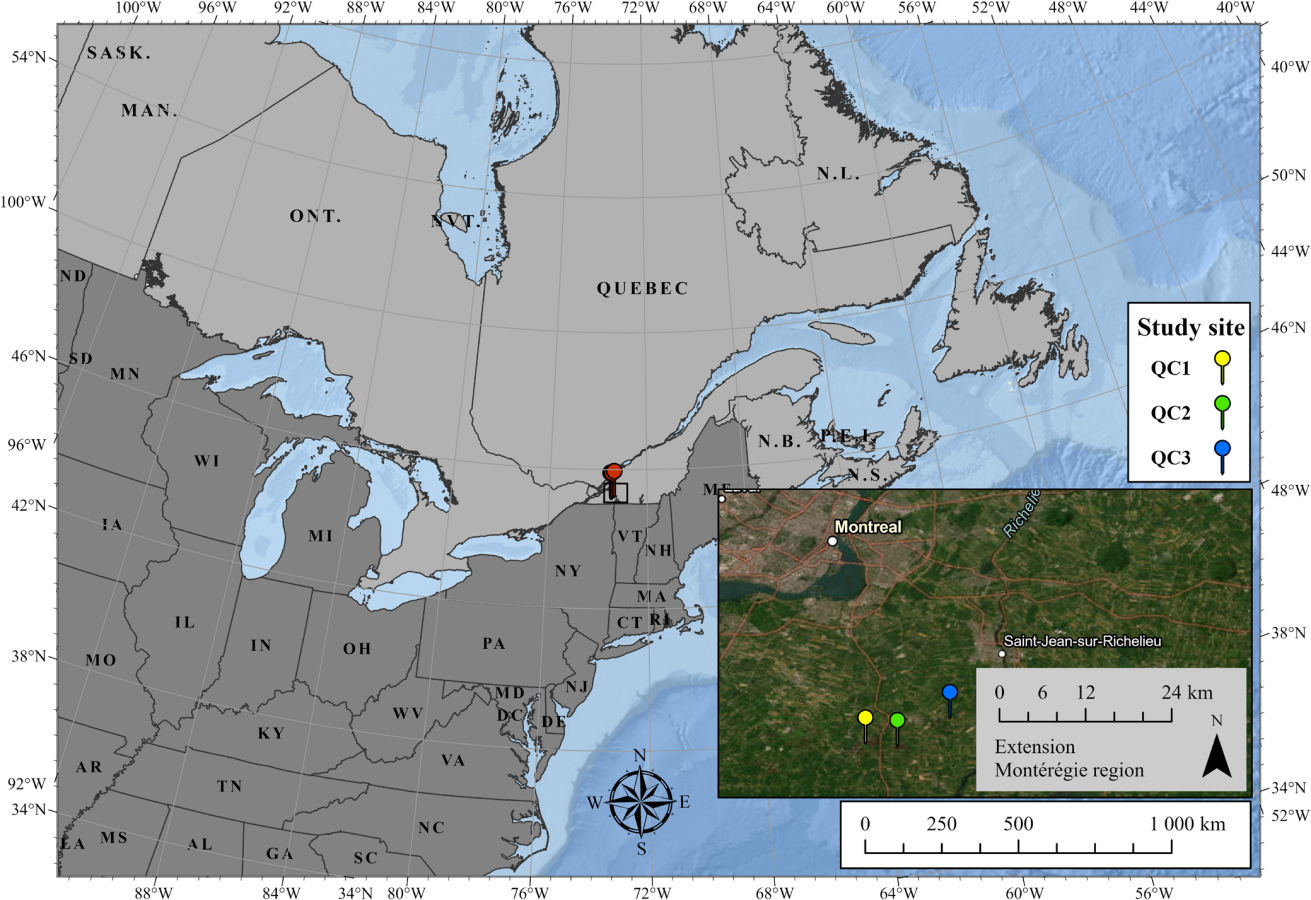
Sampling sites selected for assessing Syrphidae diversity in lettuce crops enhanced with alyssum flower strips.

Sampling was conducted in the summer of 2022 at three different commercial lettuce farms with organic soils (Histosols) in southern Quebec: the first site, covering 30.5 ha (45.145°, −73.489°); the second site, covering 20 ha (45.144°, −73.438°); and the third site, covering 16.5 ha (45.193°, −73.344°) (Figure 1). Sweet alyssum ( *L. maritima* ) was transplanted in strips along the edges of the fields at the same time as the lettuce (head lettuce; cv. Estival), ensuring synchronized growth. The alyssum was already in bloom at the time of planting (4‐week‐old transplants) to immediately attract beneficial insects, and the flowers remained in bloom throughout the lettuce growing season until harvest. At each site, one flower strip formed a border measuring 200‐m long and 1.8‐m wide. Within each strip, rows were spaced 40 cm apart, and each flower transplant within a row was spaced 30 cm apart. The control treatment consisted of a border of lettuce without flowers, located on the same site but more than 200 m from the flower border. Adjacent fields were planted with other horticultural crops commonly grown in the region, such as carrot, lettuce, and onion. Fields were irrigated and managed according to standard local organic practices.

### 2.2 Sampling

We sampled twice a week throughout the lettuce‐growing season, from 11 July to 28 August 2022 (Table S1). Since sweep‐netting remains the predominant sampling technique for syrphids (Gill and O'Neal 2015), we use this method for each sampling event. We sampled three randomly selected areas within each flower strip, performing 10 sweep net strokes (covering a 180° arc) per area. All specimens were brought back to the laboratory, frozen at −20°C for at least 24 h before being carefully transferred to Eppendorf tubes and preserved in 95% ethanol for subsequent analysis.

### 2.3 Morphological Identification

Specimens were identified to the species level based on morphological characteristics (Miranda et al. 2013; Skevington et al. 2019). A group of *Sphaerophoria* specimens was reported as a species complex. A total of 700 voucher specimens is deposited in the Ouellet‐Robert Entomological Collection (Université de Montréal, Quebec, Canada), with accession numbers QMOR 93537 to QMOR 94010 and QMOR 94611 to QMOR 94838. Specimen data are published at the Global Biodiversity Information Facility (GBIF) (Kalboussi et al. 2025).

### 2.4 DNA Barcoding and DNA Barcode Data Set Building

A subset of specimens underwent DNA barcoding, including representatives of each species and specimens of *Sphaerophoria* initially not identified to species level with morphology. DNA was extracted nondestructively using the gSYNC DNA extraction kit (Geneaid, New Taipei, Taiwan), following the manufacturer's standard protocol (but without tissue grinding) and stored at −20°C. The standardized 658 bp fragment of the mitochondrial cytochrome C oxidase subunit I (COI) of each sample was amplified using the primers LCO1490 and HCO2198 (Folmer et al. 1994). Each polymerase chain reaction (PCR) was conducted in a 20 μL reaction mixture containing 10 μL of Phire Green Hot Start II PCR Master Mix (Thermo Fisher Scientific, Waltham, MA), 2 μL each of 10 μM primers, 4 μL of template, and DNase‐free sterile water up to 20 μL. The PCR amplification was performed in a thermocycler (Bio‐Rad Laboratories Inc., Hercules, CA, USA) following the programme described by Gomez‐Polo et al. (2015). Amplified PCR fragments were sequenced with a 3730xl DNA Analyzer (Applied Biosystems) at the Genome Quebec Innovation Center (Montréal, Quebec, Canada). Sequences were edited, assembled, and checked for stop codons using Geneious Prime 2022.1.3 software (Kearse et al. 2012). Barcodes were matched to reference sequences in GenBank (National Center for Biotechnology Information; NCBI) via Basic Local Alignment Search Tool and the Barcode of Life Database (BOLD; Ratnasingham and Hebert 2013). A total of 134 identified reference DNA barcodes is available via the BOLD website under the public project name “Syrphids in Histosols of Quebec,” published (https://doi.org/10.5883/DS‐SYRPHIDS).

### 2.5 Genetic Differentiation Within the Genus *Sphaerophoria*

To assess cryptic diversity and refine species boundaries within the *Sphaerophoria* species complex, we analyzed 63 de novo COI sequences of *Sphaerophoria*, supplemented with 111 reference sequences from the BOLD database. The database search targeted 5′ COI mtDNA sequences of the *Sphaerophoria* genus from Canada, including sequences mostly identified to the species level. The sequences belonged to 11 *Sphaerophoria* taxa, with the majority of the samples belonging to *S. philanthus* (Meigen) (42 individuals), *S. scripta* (L.) (28), *Sphaerophoria* sp. (10), *S. abbreviata* Zetterstedt (6), *S. contigua* Macquart (8), *S. asymmetrica* Knutson (6), *S. bifurcata* Knutson (3), *S. longipilosa* Knutson (5), *S. brevipilosa* Knutson (1), *S. cleoae* Metcalf (1), *S. cranbrookensis* Curran (1). Sequences were aligned separately using MAFFT in Geneious Prime. We used POPART to construct haplotype networks (Leigh and Bryant 2015). This broader network provides a comprehensive view of how our sequences relate to the overall genetic diversity within the genus. Genetic diversity metrics, including nucleotide diversity (π) and the number of segregating sites, were calculated. Using the standard AMOVA approach (Excoffier et al. 1992) we assessed genetic differentiation among individuals based on the COI barcode. To determine the statistical significance of the genetic differences between haplotypes, we performed permutation tests with 10,000 permutations within the same software, providing a robust framework for evaluating the genetic diversity and structure within the complex.

The number of clusters within the total sample was investigated using the distance‐based delimitation method, ASAP (Assemble Species by Automatic Partitioning) (Puillandre et al. 2021). ASAP was chosen for its performance and simplicity in identifying sequence clusters based on pairwise distance distributions derived from the Kimura Two‐Parameter (K2P) model (Kimura 1980). This method effectively highlights potential species boundaries within the dataset (Puillandre et al. 2021). Sequences falling within an arbitrary similarity threshold (commonly 99%) (Hebert et al. 2003) were grouped into representative sequences known as Molecular Operational Units (MOTUs) (Hebert et al. 2003; de Kerdrel et al. 2020).

### 2.6 Statistical Analysis

All statistical analyses were conducted using R, version 4.5.0 (R Development Core Team 2024), using the *vegan* package (Oksanen et al. 2024). The dataset was pooled from three sampling points (sweep net passes) per strip, with sampling dates treated as replicates. Diversity indices, including Shannon (1948), Simpson (1949) and Pielou's evenness (1966), were calculated to quantify species diversity. To assess differences between treatments (with and without flower strips) within each site, we performed an ANOVA using the *stats* package. The assumptions of ANOVA were evaluated by testing normality with the Shapiro–Wilk (1965) test and homogeneity of variances with Levene's (1960) test. Community structure and patterns were visualized using nonmetric multidimensional scaling (NMDS) with the *vegan* package, with treatments and sites used as grouping variables. Species ranks were assigned using the rank() function, with higher counts corresponding to lower rank values.

The *iNEXT* package (Hsieh et al. 2016) was used to generate rarefaction and extrapolation curves to compare the syrphid diversity across three sites and two treatments: plots with alyssum flowers and control plots. Individual‐based interpolation and extrapolation of Hill numbers (*q* = 0, 1, 2) (Hill 1973) were used to assess species diversity for each site and treatment (Chao and Jost 2015). Diversity was quantified using species richness (*q* = 0), Shannon diversity (the exponential of Shannon entropy, *q* = 1), and Simpson diversity (the inverse of Simpson concentration, *q* = 2) (Chao et al. 2016). One hundred bootstrap replicates were performed, with a confidence interval level of 95%.

## 3 Results

### 3.1 Syrphid Richness

Of the 1934 syrphid specimens collected, 1852 were successfully identified to the species level, representing 13 distinct named species (Table 1). Among these, the most prevalent species collected across three sites was *Toxomerus marginatus* (Say), accounting for 69.5% of all samples (Figure S1). The second and third most abundant species in our study were *Sphaerophoria philanthus* (10.3%) and *Allograpta obliqua* (Say) (4.7%). A total of 82 *Sphaerophoria* females were identified as belonging to the *S. philanthus/asymmetrica/abbreviata* species complex, with their genetic diversity further revealed through molecular methods. The remaining species accounted for less than 4% each across all locations. In terms of trophic groups, among all collected syrphid species, 11 out of 13 identified syrphid species are aphid predators during their larval stage, representing 96.7% of the total number of individuals collected (Table 1). Of the 16 species (including unidentified MOTUs), 10 were found exclusively or predominantly (≥ 80%) in flower plots, including *Eristalis arbustorum* (L.), *Eupeodes americanus* (Wiedemann), *Melanostoma mellinum* (L.), *Syrphus rectus* Osten Sacken, *Syrphus ribesii* (L.) (all 100%), *Sphaerophoria* complex (96.2%), *Syritta pipiens* (L.) (98.2%), *Syrphus knabi* Shannon (85.7%), and *Sphaerophoria philanthus* (79.5%). Only three species showed a balanced distribution between the two treatments: *Platycheirus quadratus* (Say) (50%), *Toxomerus geminatus* (Say) (67.4%), and *T. marginatus* (75%). *Allograpta obliqua* was the only species more abundant in control plots (65.6%).

**TABLE 1:** Syrphid species found in three lettuce fields in Quebec with and without sweet alyssum flowers based on morphological and molecular analyses.

Syrphidae species	Trophic group	QC1		QC2		QC3		Total abundance	Species rank
		Flower	Control	Flower	Control	Flower	Control		
*Allograpta obliqua* (Say)	Aphidophagous	10	2	12	53	9	4	90	3
*Eristalis arbustorum* (Linnaeus)	Saprophagous	4	0	0	0	2	0	6	11
*Eupeodes americanus* (Wiedemann)	Aphidophagous	9	0	3	0	10	0	22	8
*Melanostoma mellinum* (Linnaeus)	Aphidophagous	2	0	0	0	2	0	4	12
*Platycheirus quadratus* (Say)	Aphidophagous	0	0	1	1	0	0	2	14.5
*Sphaerophoria* complex^a^ (MOTU1)	Aphidophagous	36	0	1	4	34	3	78	4
*Sphaerophoria* complex (MOTU2)	Aphidophagous	2	0	0	0	0	0	2	14.5
*Sphaerophoria* complex (MOTU3)	Aphidophagous	2	0	0	0	0	0	2	14.5
*Sphaerophoria contigua* (Macquart)	Aphidophagous	17	2	3	9	24	1	56	6
*Sphaerophoria philanthus* (Meigen)	Aphidophagous	118	28	14	7	27	6	200	2
*Syritta pipiens* (Linnaeus)	Saprophagous/detritivorous	52	1	1	0	3	0	57	5
*Syrphus knabi* (Shannon)	Aphidophagous	0	1	0	1	12	0	14	9
*Syrphus rectus* (Osten Sacken)	Aphidophagous	5	0	0	0	5	0	10	10
*Syrphus ribesii* (Linnaeus)	Aphidophagous	0	0	0	0	2	0	2	14.5
*Toxomerus geminatus* (Say)	Aphidophagous	5	0	21	13	5	2	46	7
*Toxomerus marginatus* (Say)	Aphidophagous	643	152	123	137	241	47	1343	1
Abundance		905	186	179	225	376	63	1934	

^a^ *Sphaerophoria* complex = *philanthus/asymmetrica/abbreviata*.

### 3.2 Syrphid Diversity at Lettuce Sites With and Without Alyssum Flower Strips

**FIGURE 2:**
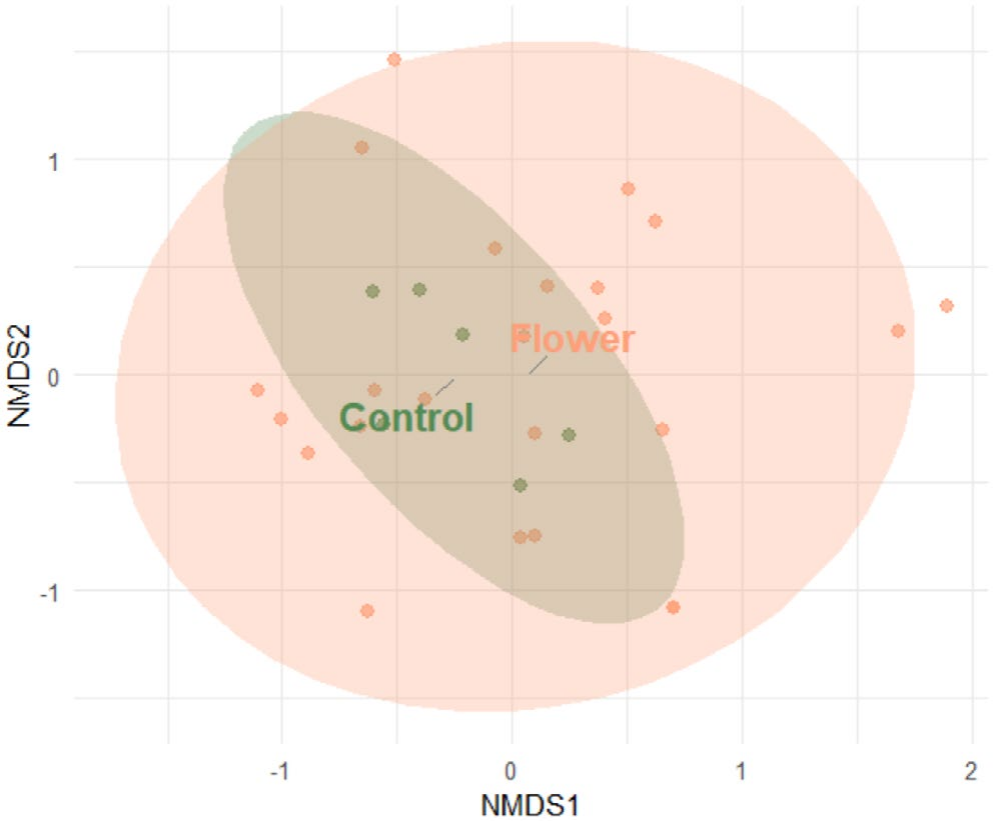
Non‐metric multidimensional scaling (NMDS) plot of Syrphidae species collected in an alyssum flower strip adjacent to lettuce (Flower) compared to a control plot with only lettuce (Control). Each point represents a sampling site and date.

**FIGURE 3:**
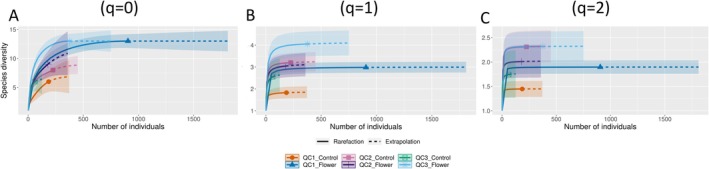
Sample‐size‐based rarefaction (solid line) and extrapolation (dotted line) curves for species richness (*q* = 0, A), Shannon‐Wiener (*q* = 1, B) and Simpson (*q* = 2, C) diversity indices, with 95% confidence intervals (shaded area), for syrphid diversity in three sampling sites, comparing those with and without alyssum flower strips.

The analysis of diversity metrics across the three sites (QC1, QC2, QC3) indicated no statistically significant differences between flower and control treatments (Richness: *F*_(1,28)_ = 0.398, *p* = 0.533; Shannon: *F*_(1,28)_ = 0.877 *p* = 0.357; Simpson: *F*_(1,28)_ = 1.025, *p* = 0.320; Pielou: *F*_(1,24)_ = 0.668, *p* = 0.422) (Table 2). This result was further supported by the NMDS analysis, which revealed considerable overlap in species composition between the flower and control treatments (*F*_(1,28)_ = 0.995, *p* = 0.385; Figure 2) and among the three sites (*F*_(2,27)_ = 1.161, *p* = 0.301; Figure S2). The rarefaction curves for flower plots (QC1_Flower, QC2_Flower, QC3_Flower) reach higher levels of species richness compared to control plots (QC1_Control, QC2_Control, QC3_Control) as the number of individuals increases (Figure 3A). The QC1_flower treatment reached a plateau when comparing the rarefaction curves, indicating that most species present in the community were captured. Similar trends are observed for Simpson and Shannon diversity (Figure 3B,C). A comparison of extrapolated species diversity shows that species richness was estimated to be higher in plots with alyssum flowers for QC3, followed by QC1 and QC2. The Shannon and Simpson diversity estimates are also highest in the flower strips for QC3 and QC1 sites (Figure 3B,C).

**TABLE 2:** Comparative diversity indices (mean ± SE) of Syrphidae at three lettuce sites with and without alyssum flower strips.

Site	Treatment	Richness	Shannon index	Simpson index	Evenness index
QC1	Control	3.50 ± 0.71^a^ *	0.65 ± 0.40^a^	0.37 ± 0.25^a^	0.51 ± 0.24^a^
	Flower	4.38 ± 2.92^a^	0.72 ± 0.31^a^	0.39 ± 0.13^a^	0.62 ± 0.19^a^
QC2	Control	5.50 ± 0.71^a^	0.78 ± 0.22^a^	0.36 ± 0.12^a^	0.46 ± 0.16^a^
	Flower	3.00 ± 2.27^a^	0.55 ± 0.43^a^	0.31 ± 0.25^a^	0.65 ± 0.27^a^
QC3	Control	4.00 ± 1.41^a^	0.76 ± 0.06^a^	0.39 ± 0.08^a^	0.58 ± 0.20^a^
	Flower	4.38 ± 3.89^a^	0.73 ± 0.64^a^	0.36 ± 0.28^a^	0.68 ± 0.24^a^

* Mean with the same letter are not significantly different (*p* > 0.05).

### 3.3 DNA Barcoding

We achieved a 75.5% barcoding success rate with 181 specimens (Table S2), meaning these sequences were of high quality and matched reference sequences in genomic databases (BOLD and GenBank) with a species‐level identification rate above 99%. Several species, including *A. obliqua* , *E. arbustorum* , *M. mellinum* , *P. quadratus* , *S. pipiens* , *S. ribesii* , *S. rectus* , *S. knabi* , *S. philanthus* , and *T. geminatus* , reached a 100% success rate in species‐level identification. *Eupeodes americanus* had a high success rate (94.1%), whereas *T. marginatus* and *S. contigua* had lower success rates (66.7% and 71%, respectively). A subset of 82 specimens, morphologically identified as belonging to the *S. philanthus/asymmetrica/abbreviata* species complex, exhibited a barcoding success rate of 52% (in terms of sequence quality). Their COI sequences could not be assigned to species with high confidence in either database (over 99% identification rate) in 90% of the cases, and incongruences in species‐level identification between the BOLD and GenBank databases were observed in 75% of the sequences. Therefore, these sequences were retained as a species complex. Notably, all sequences in this group were associated with the same Barcode Index Number (BIN) according to the BOLD database. Overall, our sequences belong to 11 BINs in the BOLD database (Table S2).

### 3.4 Genetic Differentiation Within the Genus *Sphaerophoria*

**FIGURE 4:**
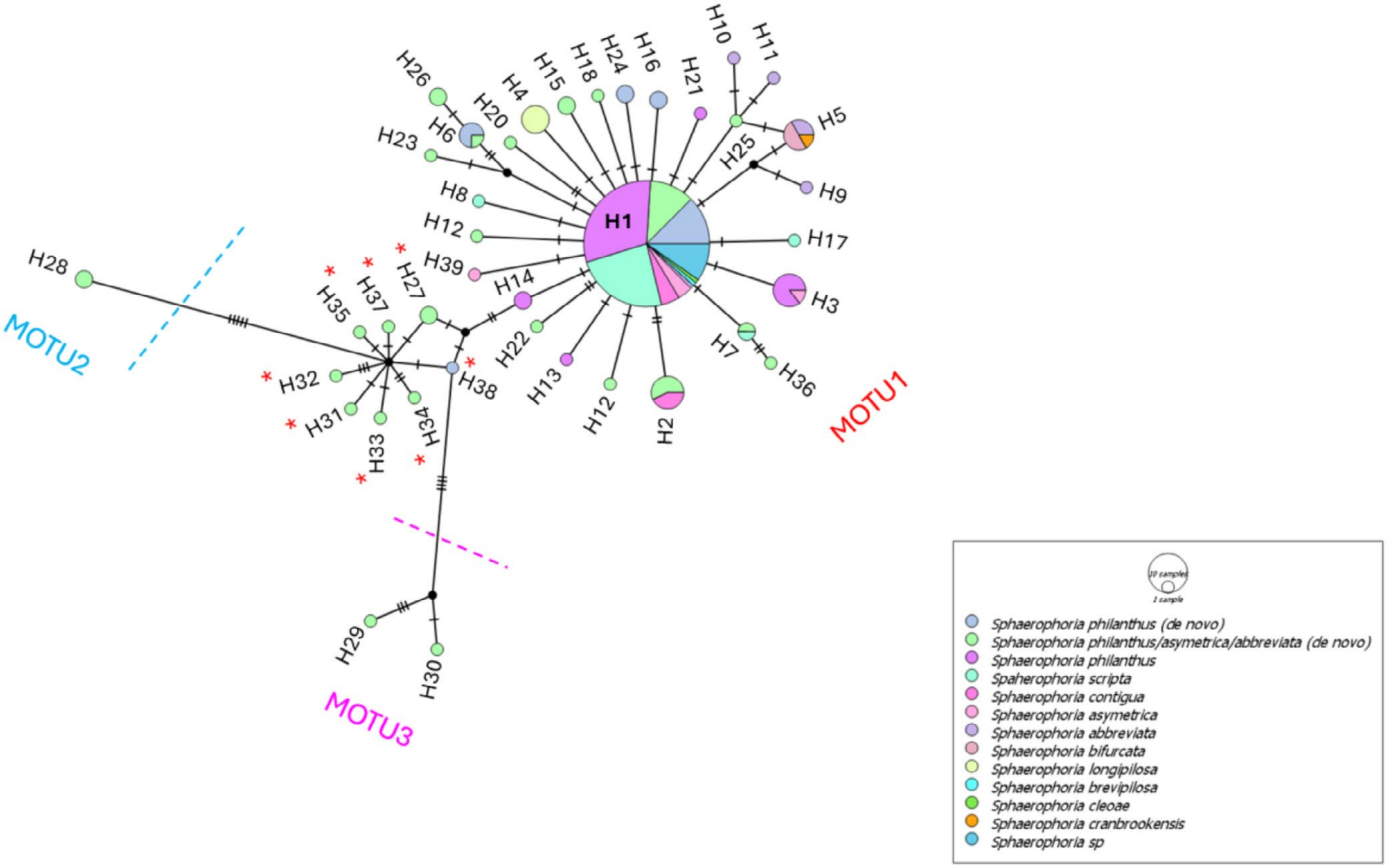
Median‐joining haplotype network inferred from *Sphaerophoria* COI DNA sequences. Each circle represents a different haplotype, its size being proportional to the number of individuals possessing it. Dashes across lines connecting the circles each indicate a pairwise nucleotide difference in haplotypes (mutations). H, haplotype; MOTU, molecular operational taxonomic unit.

The analysis of 174 COI‐DNA sequences from the genus *Sphaerophoria*, including 63 newly obtained sequences (41 females identified morphologically as *S. philanthus/asymmetrica/abbreviata* and 22 specimens as *S. philanthus* ) and 111 reference sequences, revealed 39 haplotypes with 54 segregating sites (Figure 4). The average barcode gap between *Sphaerophoria* sequences, calculated using the ASAP tool, was 1%, with p distances ranging from 0.001 to 0.01 between haplogroups. The most abundant haplotype, H1, comprised 104 of the 174 individuals (Table S3), including both reference and newly obtained sequences. Most of the remaining haplotypes differed from the central haplotype by only one to three nucleotide substitutions. Two divergent haplotype groups originating from H38 were identified: one haplotype H28, diverging by six substitutions and including two sequences related to *S. philanthus/asymmetrica/abbreviata*; the other diverging by up to seven substitutions and comprising two unique haplotypes (H29 and H30). ASAP analysis delimited between 2 and 48 MOTUs across 10 different partitions, with ASAP scores ranging from 1.5 to 14 (Table S4). The second‐best ASAP delimitation identified three distinct MOTUs: most haplotypes were clustered into MOTU1, while H28 formed MOTU2, and H29 and H30 were grouped as MOTU3.

The analysis of genetic diversity within the *Sphaerophoria* sequences revealed low nucleotide diversity (π = 0.001). Additionally, the analysis of molecular variance based on COI DNA sequences indicated no significant genetic differentiation among the analyzed *Sphaerophoria* sequences (*p* = 0.219). This suggests a lack of clear genetic structure within the dataset based on COI barcodes.

## 4 Discussion

Introducing sweet alyssum flower strips emerged as an effective strategy to boost natural aphid predator communities in this agricultural system. We found that sweet alyssum attracts a high number of syrphids, including 11 species known to be aphidophagous, accounting for 96.7% of the total abundance. Additionally, our combined morphological and molecular analysis revealed three distinct molecular operational taxonomic units (MOTUs) within the *Sphaerophoria* complex, also known to be aphidophagous. Although overall diversity metrics did not differ significantly between flower and control treatments across sites, species composition did: 10 syrphid species occurred exclusively or predominantly (≥ 80%) within the flower plots. These findings indicate that sweet alyssum strips support a more specialized syrphid community in lettuce agroecosystems. This targeted recruitment of aphidophagous species highlights the promise of floral resource provisioning for more sustainable pest management and reduced insecticide reliance.

Syrphid species collected from sweet alyssum strips in lettuce fields in Quebec are considered abundant and common in North America (Skevington et al. 2019). Several of the collected genera, including *Allograpta*, *Eupeodes*, *Melanostoma*, *Sphaerophoria*, *Syrphus*, and *Toxomerus*, are recognized for having larvae that prey on aphids (Skevington et al. 2019). Our results indicate that *T. marginatus* is the dominant species, comprising 69.5% of all individuals sampled. Interestingly, adult *T. marginatus* has been shown to exhibit a marked preference for sweet alyssum flowers (Rodríguez‐Gasol et al. 2020). This finding aligns with results from Haris‐Cypher et al. (2023) in the Northeastern United States, where 70.1% of 1447 syrphid specimens collected were *T. marginatus* , predominantly from sweet alyssum rather than buckwheat ( *Fagopyrum esculentum* Moench [Polygonaceae]) or coriander ( *Coriandrum sativum* L. [Apiaceae]). Similarly, Hogg et al. (2011) noted the dominance of *T. marginatus* in California broccoli fields managed with sweet alyssum. This affinity for sweet alyssum flowers is likely due to the alignment of *T. marginatus* 's ecological functional traits, including phenological, visual, morphological, and physiological characteristics, such as mouthpart anatomy and plant color (Violle et al. 2007; Moretti et al. 2017; Gardarin et al. 2018; Wong et al. 2019; Hatt et al. 2020). Furthermore, the life cycle of *T. marginatus* may be well synchronized with the phenology of lettuce and associated aphid populations. This synchrony could confer a competitive advantage over other syrphid species, enhancing its effectiveness as a biological control agent (Almohamad et al. 2009; Hopper et al. 2011).

*Toxomerus marginatus* is a species of syrphid that feeds on various aphid species. When reared on three different aphid species (*Aphis glycines* Matsumara, *Aphis nerii* Boyer de Fonscolombe, and *Aphis monardae* Oestlund), its developmental performance was comparable across all diets (Irvin et al. 2021). Although it is a generalist species, *T. marginatus* is not among the most voracious aphid predators when compared to other syrphid species. For instance, in a study involving the aphid *N. ribisnigri* , *T. marginatus* consumed nearly four times fewer aphids over its larval stage than the syrphid *Eupeodes fumipennis* (Thomson) (132 aphids for *T. marginatus* vs. 507 for *E. fumipennis* ) (Hopper et al. 2011). Nevertheless, compared to other aphidophagous predators such as Cecidomyiidae (Diptera), Coccinellidae (Coleoptera) and spiders (Aranea), Syrphidae (including *T. marginatus* ) are considered to be among the most voracious natural enemies of *N. ribisnigri* (Hopper et al. 2011); they play a key role in aphid population regulation.

For more than 20 years, alyssum has been planted as an insectary plant in organic lettuce fields in California, where it has been shown to enhance the abundance of syrphid larvae in lettuce (B. Chaney 2003; Smith and Chaney 2007; Smith et al. 2008) and reduce aphid densities (Hogg et al. 2011). The richness of predatory syrphid species observed in sweet alyssum flower strips within lettuce fields in Quebec reveals both notable similarities and differences when compared to the findings of Smith and Chaney (2007) in California's lettuce fields. In both regions, *T. marginatus* emerges as the dominant species, accounting for 69.5% of individuals in Quebec and 39% in California. *Sphaerophoria* species comprise 17.5% of individuals in Quebec and 13% in California, although the specific species differ between the two locations. *Sphaerophoria contigua* is commonly observed in the lettuce fields of both California (Smith et al. 2008) and Quebec. *Sphaerophoria philanthus* is the second most abundant species in Quebec but has not been collected in California. In contrast, *Sphaerophoria sulfuripes* (Thomson) and *Sphaerophoria pyrrhina* (Bigot) were found exclusively in the California study. The genus *Allograpta* is present in both regions, yet it exhibits a higher relative abundance in California (10%) compared to Quebec (4.8%). Significant differences are also evident in the composition of *Platycheirus* species; for instance, *Platycheirus stegnus* (Say) is the second most abundant species in California (27%) but is absent in Quebec, whereas *Platycheirus quadratus* is rare (less than 1%) in the California study. The genus *Syrphus* is more diverse in Quebec, with three species, *S. rectus* , *S. ribesii* , and *S. knabi* , not recorded in California. Furthermore, certain predatory species such as *E. americanus* , *M. mellinum* , and *T. geminatus* are uniquely found in Quebec samples. Conversely, species such as *Toxomerus occidentalis* (Curran), *Eupeodes volucris* (Osten Sacken), and *Scaeva pyrastri* (L.) are exclusively collected in the California study. These differences in species richness and relative abundance likely reflect climatic, ecological, and biogeographical variations between eastern and western North America, as well as methodological differences between the studies.

The analysis of diversity metrics, including the Simpson and Shannon indices, revealed no statistically significant differences between flower and control treatments at the three study sites. One plausible explanation for these results lies in the high dispersal ability of syrphids, which can travel over 100 m to 1 km (Wratten et al. 2003; Haenke et al. 2009). This mobility could have led to a homogenization of community composition between treatments, especially when the spatial separation between flower strips and control plots was limited (at least 200 m). Moreover, previous research in lettuce agroecosystems has shown that manipulating the spatial distribution of floral resources influences syrphid behavior, suggesting that their mobility can blur the distinctions between treatments (Gillespie et al. 2011). Pollinator species *S. pipiens* and *E. arbustorum* were exclusively present in flower strips. Studies have shown that while flower strips may not significantly increase syrphid species richness, they often enhance syrphid abundance (Bianchi et al. 2006; Scheper et al. 2015; Killewald et al. 2024). Slight increases in species evenness suggest that flower strips can foster a more balanced syrphid assemblage, which may enhance ecosystem resilience (Hillebrand et al. 2008).

The plateau observed in the rarefaction curve for flower plots indicates sampling sufficiency, suggesting that most species of the community were likely captured, thereby enabling an efficient estimation of true diversity (Magurran 2013). At QC2, control plots unexpectedly exhibited higher richness and diversity than flower plots. Such site‐specific reversals are not uncommon (Killewald et al. 2024), although the underlying causes remain unclear and may be attributed to local habitat characteristics or the broader landscape context (Scheper et al. 2015). Overall, our findings suggest that flower strips may support a more balanced and potentially more resilient syrphid community. However, definitive conclusions about their efficacy remain premature due to limited replication, single‐season sampling, and the absence of aphid population assessments on lettuce in the field. Robust comparisons will require expanded experimental designs with multi‐year data to account for natural variation in syrphid populations (Tschumi, Albrecht, Bärtschi, et al. 2016; Killewald et al. 2024).

The syrphid genetic diversity sampled in this study was used to create a DNA barcode library, integrating both morphological and molecular identification. This database will be essential for future syrphid larval stage identifications in the context of biological control. However, morphological and molecular identification proved to be challenging within *Sphaerophoria*. This difficulty was exacerbated by the predominance of female specimens, which lack distinctive secondary sexual characteristics and morphologically resemble one another (Haarto and Kerppola 2007; Van Veen 2010). Even males within *Sphaerophoria* exhibit subtle morphological differences, further complicating species identification (Bartsch et al. 2009). Previous studies have often limited *Sphaerophoria* identification to the genus level due to these challenges (W. E. Chaney 1998; Hogg et al. 2011; Haris‐Cypher et al. 2023; Moquet et al. 2018). These findings highlight the limitations of relying solely on morphological traits to capture species richness, particularly in groups with cryptic species.

We used the mitochondrial cytochrome c oxidase subunit I gene (COI mtDNA) as a DNA barcoding marker to delineate species boundaries within the *Sphaerophoria* species complex. The barcoding success rate for the *Sphaerophoria* species complex was notably low compared to other genera. In 90% of cases, identification of *Sphaerophoria* sequences could only be made to the genus level in at least one of the two queried databases (BOLD and NCBI). Furthermore, 75% of *Sphaerophoria* sequences showed discrepancies in species‐level identification between these databases. Notably, all of our *Sphaerophoria* sequences were assigned to a single Barcode Index Number (BIN), BOLD:AAA7374, which is one of only two BINs reported for this genus in Canada, according to the BOLD database. The BOLD genomic database currently contains 2586 published records for *Sphaerophoria* globally, with species names assigned to only 637 of these records (https://v4.boldsystems.org/index.php/Public_SearchTerms, as of November, 2024). The majority of records are still categorized at the genus level, highlighting the global challenges in accurately identifying and clustering species within this group.

In this study, we identified 39 distinct haplotypes among the 174 *Sphaerophoria* sequences analyzed from specimens collected in Canada. Analysis of the COI gene revealed relatively low overall genetic diversity, with a barcode gap of only 1% between sequences, which is below the typical threshold of 2% (Hebert et al. 2003). This suggests that the genetic variation within species is nearly as high as the variation between species. Notably, network analysis revealed three distinct groups, delimited into three MOTUs based on one of the most prevalent partitions of ASAP. We observed significant haplotype sharing between our individuals and those identified as different species in the BOLD database based on the COI gene, with most being grouped in the central haplotype H1. This finding could be interpreted by various hypotheses. First, misidentifications in genomic databases might lead to erroneous species classifications. Second, the COI gene alone may not be sufficient to differentiate species that have recently diverged (recent speciation), as the genetic differences may be too subtle for this marker to detect (Funk and Omland 2003; Kim and Han 2022; Zhang and Bu 2022). Third, hybridization between populations could be occurring, which would also go undetected by the COI gene, suggesting that the individuals analyzed may belong to a genetically homogeneous population. This homogeneity could result from high gene flow between geographically close regions (Raymond et al. 2013), which reduces opportunities for genetic differentiation (Bessette et al. 2022). Additionally, the second and third MOTUs consist exclusively of sequences belonging to the *Sphaerophoria philanthus/asymmetrica/abbreviata* complex. This clustering of sequences from a single location, with no shared haplotypes with other samples, may be attributed to genetic drift, particularly given the small number of individuals analyzed (Star and Spencer 2013). Genetic drift can lead to random fluctuations in allele frequencies, resulting in genetically distinct groups that do not share common haplotypes with others (Hedrick et al. 2006; Milankov et al. 2008). Alternatively, this could indicate the presence of a cryptic species that has existed for a long time but has never been identified using molecular tools. These hypotheses reflect ambiguity in species‐level delimitation within *Sphaerophoria* based solely on the COI gene (Popović et al. 2015; Gojković et al. 2020), which also aligns with other studies showing low COI resolution for syrphid species differentiation (Kim and Han 2022) and some other insect genera (Moritz and Cicero 2004; Milankov et al. 2008; Virgilio et al. 2010).

For our research aimed at identifying promising taxa for conservation biological control, characterizing the *Sphaerophoria* to the species complex level is sufficient, as all three putative species are recognized aphid predators (Bugg et al. 2008; Skevington et al. 2019). However, for a deeper understanding of the genetic structure and species boundaries within the *Sphaerophoria* complex, additional analyses on a larger sample are necessary. This should include a taxonomic revision and the use of complementary genetic markers or approaches (Mengual et al. 2008; Young et al. 2016).

Our study provides valuable insights into the diversity of syrphids within sweet alyssum flower strips cultivated in Quebec lettuce fields. Our results highlight the prevalence of aphid predators among the dominant taxa, suggesting that integrating sweet alyssum flower strips could effectively enhance conservation biological control strategies in Quebec's lettuce production. Furthermore, we have established a molecular database containing DNA sequences from syrphids sampled in these fields. This database supports our broader project in conservation biological control, facilitating larval stage identification and surveys of syrphid biodiversity in Quebec and neighboring regions.

## Author Contributions

**Malek Kalboussi:** conceptualization (lead), data curation (lead), formal analysis (lead), investigation (lead), methodology (lead), writing – original draft (lead). **Alice Dabrowski:** investigation (supporting), validation (equal), writing – review and editing (supporting). **Andrew D. Young:** validation (supporting), writing – review and editing (supporting). **Annie‐Ève Gagnon:** conceptualization (equal), funding acquisition (lead), investigation (equal), methodology (equal), project administration (equal), supervision (equal), validation (equal), writing – review and editing (equal). **Colin Favret:** conceptualization (equal), methodology (equal), project administration (equal), project administration (equal), supervision (equal), supervision (equal), validation (equal), validation (equal), writing – review and editing (equal), writing – review and editing (equal).

## Conflicts of Interest

The authors declare no conflicts of interest.

## Supporting information


**Data S1:** ece372145‐sup‐0001‐Supinfo.docx.

## Data Availability

A total of 700 voucher specimens are deposited in the Ouellet‐Robert Entomological Collection at the Université de Montréal, at the Institut de recherche en biologie végétale (IRBV), Centre sur la biodiversité of the University, Quebec, Canada, with accession numbers QMOR 93537–QMOR 94010 and QMOR 94611–QMOR 94838. Specimen data are published in the Global Biodiversity Information Facility (GBIF) (Kalboussi et al. 2025; https://specimenpub.org/collection/collection_7/). 134 DNA‐barcoded voucher specimens are available via the BOLD website under the public project name “Syrphids in Histosols of Quebec,” published (https://doi.org/10.5883/DS‐SYRPHIDS), and conducted using BOLD v4.

## References

[ece372145-bib-0001] Agriculture and Agri‐Food Canada (AAFC) . 2021. “Crop Profile for Field Lettuce and Spinach in Canada.” https://publications.gc.ca/collections/collection_2023/aac‐aafc/A118‐10‐41‐2021‐eng.pdf.

[ece372145-bib-0002] Albrecht, M. , D. Kleijn , N. M. Williams , et al. 2020. “The Effectiveness of Flower Strips and Hedgerows on Pest Control, Pollination Services and Crop Yield: A Quantitative Synthesis.” Ecology Letters 23, no. 10: 1488–1498. 10.1111/ele.13576. 32808477 PMC7540530

[ece372145-bib-0003] Almohamad, R. , F. J. Verheggen , and E. Haubruge . 2009. “Searching and Oviposition Behavior of Aphidophagous Hoverflies (Diptera: Syrphidae): A Review.” Biotechnology, Agronomy, Society and Environment 13, no. 3: 467–481.

[ece372145-bib-0004] Ambrosino, M. D. , J. M. Luna , P. C. Jepson , and S. D. Wratten . 2006. “Relative Frequencies of Visits to Selected Insectary Plants by Predatory Hoverflies (Diptera: Syrphidae), Other Beneficial Insects, and Herbivores.” Environmental Entomology 35, no. 2: 394–400. 10.1603/0046-225X-35.2.394.

[ece372145-bib-0005] Badenes‐Pérez, F. R. 2019. “Trap Crops and Insectary Plants in the Order Brassicales.” Annals of the Entomological Society of America 112, no. 4: 318–329. 10.1093/aesa/say043.

[ece372145-bib-0006] Barcode of Life Database (BOLD) v4 . 2024. https://v4.boldsystems.org/index.php/Public_SearchTerms.

[ece372145-bib-0007] Bartsch, H. , E. Binkiewicz , A. Rådén , and E. Nasibov . 2009. National nyckeln till Sveriges flora och fauna. Diptera: Syrphidae: Syrphinae. ArtDatabanken, SLU.

[ece372145-bib-0008] Bessette, M. , D. T. Ste‐Croix , J. Brodeur , B. Mimee , and A.‐È. Gagnon . 2022. “Population Genetic Structure of the Carrot Weevil ( *Listronotus oregonensis* ) in North America.” Evolutionary Applications 15, no. 2: 300–315. 10.1111/eva.13343. 35233249 PMC8867704

[ece372145-bib-0009] Bianchi, F. J. J. A. , C. J. H. Booij , and T. Tscharntke . 2006. “Sustainable Pest Regulation in Agricultural Landscapes: A Review on Landscape Composition, Biodiversity and Natural Pest Control.” Proceedings of the Royal Society B: Biological Sciences 273, no. 1595: 1715–1727. 10.1098/rspb.2006.3530. PMC1634792 16790403

[ece372145-bib-0010] Brennan, E. B. 2013. “Agronomic Aspects of Strip Intercropping Lettuce With Alyssum for Biological Control of Aphids.” Biological Control 65, no. 3: 302–311. 10.1016/j.biocontrol.2013.03.017.

[ece372145-bib-0011] Brennan, E. B. 2016. “Agronomy of Strip Intercropping Broccoli With Alyssum for Biological Control of Aphids.” Biological Control 97: 109–119. 10.1016/j.biocontrol.2016.02.015.

[ece372145-bib-0012] Bugg, R. L. , R. G. Colfer , W. E. Chaney , H. A. Smith , and J. Cannon . 2008. Flower Flies (Syrphidae) and Other Biological Control Agents for Aphids in Vegetable Crops. University of California, Agriculture and Natural Resources. 10.3733/ucanr.8285.

[ece372145-bib-0013] Campbell, A. J. , J. C. Biesmeijer , V. Varma , and F. L. Wäckers . 2012. “Realising Multiple Ecosystem Services Based on the Response of Three Beneficial Insect Groups to Floral Traits and Trait Diversity.” Basic and Applied Ecology 13, no. 4: 363–370. 10.1016/j.baae.2012.04.003.

[ece372145-bib-0014] Chaney, B. 2003. “Insect Management for Central Coast Lettuce, California Lettuce.” Research Board Reports, pp. 165–181.

[ece372145-bib-0015] Chaney, W. E. 1998. “Biological Control of Aphids in Lettuce Using In‐Field Insectaries.” In Enhancing Biological Control: Habitat Management to Promote Natural Enemies of Arthropod Pests, edited by C. H. Pickett and R. L. Bugg , 73–83. University of California Press.

[ece372145-bib-0016] Chao, A. , and L. Jost . 2015. “Estimating Diversity and Entropy Profiles via Discovery Rates of New Species.” Methods in Ecology and Evolution 6, no. 8: 873–882. 10.1111/2041-210X.12349.

[ece372145-bib-0017] Chao, A. , K. H. Ma , and T. C. Hsieh . 2016. “iNEXT (iNterpolation and EXTrapolation) Online: Software for Interpolation and Extrapolation of Species Diversity.” Program and User's Guide. http://chao.stat.nthu.edu.tw/wordpress/software_download/inext‐online/.

[ece372145-bib-0018] Colley, M. R. , and J. M. Luna . 2000. “Relative Attractiveness of Potential Beneficial Insectary Plants to Aphidophagous Hoverflies (Diptera: Syrphidae).” Environmental Entomology 29, no. 5: 1054–1059. 10.1603/0046-225X-29.5.1054.

[ece372145-bib-0019] de Kerdrel, G. A. , J. C. Andersen , S. R. Kennedy , R. Gillespie , and H. Krehenwinkel . 2020. “Rapid and Cost‐Effective Generation of Single Specimen Multilocus Barcoding Data From Whole Arthropod Communities by Multiple Levels of Multiplexing.” Scientific Reports 10, no. 1: 78. 10.1038/s41598-019-54927-z. 31919378 PMC6952404

[ece372145-bib-0020] Díaz, B. M. , M. Muñiz , L. Barrios , and A. Fereres . 2007. “Temperature Thresholds and Thermal Requirements for Development of *Nasonovia ribisnigri* (Hemiptera: Aphididae).” Environmental Entomology 36, no. 4: 681–688. 10.1603/0046-225x(2007)36[681:ttatrf]2.0.co;2. 17716458

[ece372145-bib-0021] Dumotier, J. , J. Brodeur , and A.‐È. Gagnon . 2024. “Trophic Impacts of a Rolled‐Rye Cover Crop on the Lettuce Aphid ( *Nasonovia ribisnigri* ) and Associated Insect Fauna in Cultivated Histosols.” Entomologia Experimentalis et Applicata 172, no. 3: 215–228. 10.1111/eea.13402.

[ece372145-bib-0022] Dunn, L. , M. Lequerica , C. R. Reid , and T. Latty . 2020. “Dual Ecosystem Services of Syrphid Flies (Diptera: Syrphidae): Pollinators and Biological Control Agents.” Pest Management Science 76, no. 6: 1973–1979. 10.1002/ps.5807. 32115861

[ece372145-bib-0023] Eilenberg, J. , A. Hajek , and C. Lomer . 2001. “Suggestions for Unifying the Terminology in Biological Control.” Biocontrol 46, no. 4: 387–400. 10.1023/A:1014193329979.

[ece372145-bib-0024] Excoffier, L. , P. E. Smouse , and J. M. Quattro . 1992. “Analysis of Molecular Variance Inferred From Metric Distances Among DNA Haplotypes: Application to Human Mitochondrial DNA Restriction Data.” Genetics 131, no. 2: 479–491. 10.1093/genetics/131.2.479. 1644282 PMC1205020

[ece372145-bib-0025] Folmer, O. , M. Black , W. Hoeh , R. Lutz , and R. Vrijenhoek . 1994. “DNA Primers for Amplification of Mitochondrial Cytochrome c Oxidase Subunit I From Diverse Metazoan Invertebrates.” Molecular Marine Biology and Biotechnology 3, no. 5: 294–299. 7881515

[ece372145-bib-0026] Fountain, M. T. 2022. “Impacts of Wildflower Interventions on Beneficial Insects in Fruit Crops: A Review.” Insects 13, no. 3: 304. 10.3390/insects13030304. 35323602 PMC8955123

[ece372145-bib-0027] Funk, D. J. , and K. E. Omland . 2003. “Species‐Level Paraphyly and Polyphyly: Frequency, Causes, and Consequences, With Insights From Animal Mitochondrial DNA.” Annual Review of Ecology, Evolution, and Systematics 34, no. 1: 397–423. 10.1146/annurev.ecolsys.34.011802.132421.

[ece372145-bib-0028] Gardarin, A. , M. Plantegenest , A. Bischoff , and M. Valantin‐Morison . 2018. “Understanding Plant–Arthropod Interactions in Multitrophic Communities to Improve Conservation Biological Control: Useful Traits and Metrics.” Journal of Pest Science 91, no. 3: 943–955. 10.1007/s10340-018-0958-0.

[ece372145-bib-0029] Gill, K. A. , and M. E. O'Neal . 2015. “Survey of Soybean Insect Pollinators: Community Identification and Sampling Method Analysis.” Environmental Entomology 44, no. 3: 488–498. 10.1093/ee/nvv001. 26313954

[ece372145-bib-0030] Gillespie, M. , S. Wratten , R. Sedcole , and R. Colfer . 2011. “Manipulating Floral Resources Dispersion for Hoverflies (Diptera: Syrphidae) in a California Lettuce Agro‐Ecosystem.” Biological Control 59, no. 2: 215–220. 10.1016/j.biocontrol.2011.07.010.

[ece372145-bib-0031] Gojković, N. , L. Francuski , J. Ludoški , and V. Milankov . 2020. “DNA Barcode Assessment and Population Structure of Aphidophagous Hoverfly *Sphaerophoria scripta* : Implications for Conservation Biological Control.” Ecology and Evolution 10, no. 17: 9428–9443. 10.1002/ece3.6631. 32953072 PMC7487226

[ece372145-bib-0032] Gomez‐Polo, P. , O. Alomar , C. Castañé , J. G. Lundgren , J. Piñol , and N. Agustí . 2015. “Molecular Assessment of Predation by Hoverflies (Diptera: Syrphidae) in Mediterranean Lettuce Crops: Molecular Assessment of Predation by Hoverflies in Lettuce.” Pest Management Science 71, no. 9: 1219–1227. 10.1002/ps.3910. 25236922

[ece372145-bib-0033] Government of Canada . 2023. “Lettuce (*Lactuca sativa* L.) Cultivation Statistics.” https://agriculture.canada.ca/en/sector/horticulture/reports/statistical‐overview‐canadian‐field‐vegetable‐industry‐2023.

[ece372145-bib-0034] Haarto, A. , and S. Kerppola . 2007. Suomen kukkakärpäset ja lähialueiden lajeja. Suomen ympäristöministeriö, 647 pp.

[ece372145-bib-0035] Haenke, S. , B. Scheid , M. Schaefer , T. Tscharntke , and C. Thies . 2009. “Increasing Syrphid Fly Diversity and Density in Sown Flower Strips Within Simple vs. Complex Landscapes.” Journal of Applied Ecology 46, no. 5: 1106–1114. 10.1111/j.1365-2664.2009.01685.x.

[ece372145-bib-0036] Haris‐Cypher, A. , C. Roman , G. Higgins , et al. 2023. “A Field Survey of Syrphid Species and Adult Densities on Annual Flowering Plants in the Northeastern United States.” Environmental Entomology 52, no. 2: 175–182. 10.1093/ee/nvad016. 36800248

[ece372145-bib-0037] Hatt, S. , F. Francis , Q. Xu , S. Wang , and N. Osawa . 2020. “Perennial Flowering Strips for Conservation Biological Control of Insect Pests: From Picking and Mixing Flowers to Tailored Functional Diversity.” In Integrative Biological Control, vol. 20, edited by Y. Gao , H. M. T. Hokkanen , and I. Menzler‐Hokkanen , 57–71. Springer International Publishing. 10.1007/978-3-030-44838-7_4.

[ece372145-bib-0038] Hebert, P. D. N. , A. Cywinska , S. L. Ball , and J. R. deWaard . 2003. “Biological Identifications Through DNA Barcodes.” Proceedings of the Royal Society of London, Series B: Biological Sciences 270, no. 1512: 313–321. 10.1098/rspb.2002.2218. PMC1691236 12614582

[ece372145-bib-0039] Hedrick, P. W. , R. N. Lee , and C. R. Hurt . 2006. “The Endangered Sonoran Topminnow: Examination of Species and ESUs Using Three mtDNA Genes.” Conservation Genetics 7, no. 4: 483–492. 10.1007/s10592-005-9058-9.

[ece372145-bib-0040] Hickman, J. M. , and S. D. Wratten . 1996. “Use of *Phelia tanacetifoli*a Strips to Enhance Biological Control of Aphids by Overfly Larvae in Cereal Fields.” Journal of Economic Entomology 89, no. 4: 832–840. 10.1093/jee/89.4.832.

[ece372145-bib-0041] Hill, M. O. 1973. “Diversity and Evenness: A Unifying Notation and Its Consequences.” Ecology 54, no. 2: 427–432. 10.2307/1934352.

[ece372145-bib-0042] Hillebrand, H. , D. M. Bennett , and M. W. Cadotte . 2008. “Consequences of Dominance: A Review of Evenness Effects on Local and Regional Ecosystem Processes.” Ecology 89, no. 6: 1510–1520. 10.1890/07-1053.1. 18589516

[ece372145-bib-0044] Hogg, B. N. , E. H. Nelson , and K. M. Daane . 2023. “A Comparison of Candidate Banker Plants for Management of Pests in Lettuce.” Environmental Entomology 52, no. 3: 379–390. 10.1093/ee/nvad029. 37043620

[ece372145-bib-0045] Hogg, B. N. , E. H. Nelson , N. J. Mills , and K. M. Daane . 2011. “Floral Resources Enhance Aphid Suppression by a Hoverfly: Flowers Enhance Aphid Suppression by a Hoverfly.” Entomologia Experimentalis et Applicata 141, no. 2: 138–144. 10.1111/j.1570-7458.2011.01174.x.

[ece372145-bib-0046] Holman, J. 2008. “BOOK REVIEW: Blackman R.L. & Eastop V.F.: Aphids on the World's Herbaceous Plants and Shrubs.” European Journal of Entomology 105, no. 1: 164. 10.14411/eje.2008.024.

[ece372145-bib-0047] Hopper, J. V. , E. H. Nelson , K. M. Daane , and N. J. Mills . 2011. “Growth, Development and Consumption by Four Syrphid Species Associated With the Lettuce Aphid, *Nasonovia ribisnigri*, in California.” Biological Control 58, no. 3: 271–276. 10.1016/j.biocontrol.2011.03.017.

[ece372145-bib-0048] Hsieh, T. C. , K. H. Ma , and A. Chao . 2016. “iNEXT: An R Package for Rarefaction and Extrapolation of Species Diversity (Hill Numbers).” Methods in Ecology and Evolution 7, no. 12: 1451–1456. 10.1111/2041-210X.12613.

[ece372145-bib-0049] Irvin, N. A. , C. Pierce , and M. S. Hoddle . 2021. “Evaluating the Potential of Flowering Plants for Enhancing Predatory Hoverflies (Syrphidae) for Biological Control of *Diaphorina citri* (Liviidae) in California.” Biological Control 157: 104574. 10.1016/j.biocontrol.2021.104574.

[ece372145-bib-0050] Kalboussi, M. , A. Dabrowski , and C. Audette . 2025. “Hover Flies (Diptera: Syrphidae) From Sweet Alyssum Flower Strips in Quebec Lettuce Fields.” Collection 7: 2. https://specimenpub.org/collection/collection_7/.

[ece372145-bib-0051] Kearse, M. , R. Moir , A. Wilson , et al. 2012. “Geneious Basic: An Integrated and Extendable Desktop Software Platform for the Organization and Analysis of Sequence Data.” Bioinformatics 28, no. 12: 1647–1649. 10.1093/bioinformatics/bts199. 22543367 PMC3371832

[ece372145-bib-0052] Killewald, M. F. , A. C. Costamagna , Y. Lawley , R. H. Gulden , and J. Gibbs . 2024. “Floral Strips Adjacent to Manitoba Crop Fields Attract Beneficial Insects Shortly After Establishment Regardless of Management Type or Landscape Context.” Agricultural and Forest Entomology 26, no. 1: 18–37. 10.1111/afe.12595.

[ece372145-bib-0053] Kim, C.‐O. , and H.‐Y. Han . 2022. “Clarifying the Identity of Two Resembling Hoverfly Species, *Betasyrphus serarius* and *B. nipponensis* (Diptera: Syrphidae: Syrphini), Based on Morphology and DNA Barcoding.” Journal of Asia‐Pacific Entomology 25, no. 2: 101914. 10.1016/j.aspen.2022.101914.

[ece372145-bib-0054] Kimura, M. 1980. “A Simple m. Ethod for Estimating Evolutionary Rates of Base Substitutions Through Comparative Studies of Nucleotide Sequences.” Journal of Molecular Evolution 16, no. 2: 111–120. 10.1007/BF01731581. 7463489

[ece372145-bib-0055] Klarich, K. L. , N. C. Pflug , E. M. DeWald , et al. 2017. “Occurrence of Neonicotinoid Insecticides in Finished Drinking Water and Fate During Drinking Water Treatment.” Environmental Science & Technology Letters 4, no. 5: 168–173. 10.1021/acs.estlett.7b00081.

[ece372145-bib-0056] Köneke, A. , R. Uesugi , A. Herz , et al. 2023. “Effects of Wheat Undersowing and Sweet Alyssum Intercropping on Aphid and Flea Beetle Infestation in White Cabbage in Germany and Japan.” Journal of Plant Diseases and Protection 130, no. 3: 619–631. 10.1007/s41348-023-00730-y.

[ece372145-bib-0057] Kordbacheh, F. , M. Liebman , and M. Harris . 2020. “Strips of Prairie Vegetation Placed Within Row Crops Can Sustain Native Bee Communities.” PLoS One 15, no. 10: e0240354. 10.1371/journal.pone.0240354. 33120405 PMC7595394

[ece372145-bib-0058] Landis, D. A. , S. D. Wratten , and G. M. Gurr . 2000. “Habitat Management to Conserve Natural Enemies of Arthropod Pests in Agriculture.” Annual Review of Entomology 45, no. 1: 175–201. 10.1146/annurev.ento.45.1.175. 10761575

[ece372145-bib-0059] Leigh, J. W. , and D. Bryant . 2015. “ popart: Full‐Feature Software for Haplotype Network Construction.” Methods in Ecology and Evolution 6, no. 9: 1110–1116. 10.1111/2041-210X.12410.

[ece372145-bib-0060] Levene, H. 1960. “Robust Tests for Equality of Variances.” In Contributions to Probability and Statistics: Essays in Honor of Harold Hotelling, edited by I. Olkin , 278–292. Stanford University Press.

[ece372145-bib-0061] Magurran, A. E. 2013. Measuring Biological Diversity. John Wiley & Sons.

[ece372145-bib-0062] Malaj, E. , K. Liber , and C. A. Morrissey . 2020. “Spatial Distribution of Agricultural Pesticide Use and Predicted Wetland Exposure in the Canadian Prairie Pothole Region.” Science of the Total Environment 718: 134765. 10.1016/j.scitotenv.2019.134765. 31843311

[ece372145-bib-0063] MAPAQ . 2020. “Plan de surveillance des résidus de pesticides dans les fruits et légumes frais issus de la culture conventionnelle vendus au Québec.” https://www.mapaq.gouv.qc.ca/fr/Publications/PlanSurveillance_Residus_Pesticides_Fruits_Legumes_2018‐2019_Accessible.pdf.

[ece372145-bib-0064] Martinez, E. , C. A. Marcillo‐Paguay , E. G. Revelo‐Gomez , M. Cuervo , and E. P. Igua‐Urbano . 2024. “Effect of Flowering Strips in Associated Broccoli and Lettuce Crops on Increasing Land Use Efficiency.” Sustainability 16, no. 11: 4436. 10.3390/su16114436.

[ece372145-bib-0065] Mateos‐Fierro, Z. , M. T. Fountain , M. P. D. Garratt , K. Ashbrook , and D. B. Westbury . 2021. “Active Management of Wildflower Strips in Commercial Sweet Cherry Orchards Enhances Natural Enemies and Pest Regulation Services.” Agriculture, Ecosystems & Environment 317: 107485. 10.1016/j.agee.2021.107485.

[ece372145-bib-0066] Matthews, G. 2017. “Aphids as Crop Pests.” In Outlooks on Pest Management, 28(5), edited by H. F. van Emden and H. Richard , 235. Research Information Ltd. 10.1564/v28_oct_09.

[ece372145-bib-0068] Mengual, X. , G. Stahls , and S. Rojo . 2008. “Molecular Phylogeny of *Allograpta* (Diptera, Syrphidae) Reveals Diversity of Lineages and Non‐Monophyly of Phytophagous Taxa.” Molecular Phylogenetics and Evolution 49, no. 3: 715–727. 10.1016/j.ympev.2008.09.011. 18848633

[ece372145-bib-0069] Milankov, V. , G. Ståhls , J. Stamenković , and A. Vujić . 2008. “Genetic Diversity of Populations of *Merodon Aureus* and *M. cinereus* Species Complexes (Diptera, Syrphidae): Integrative Taxonomy and Implications for Conservation Priorities on the Balkan Peninsula.” Conservation Genetics 9, no. 5: 1125–1137. 10.1007/s10592-007-9426-8.

[ece372145-bib-0070] Miranda, G. F. G. , A. D. Young , M. M. Locke , S. A. Marshall , J. H. Skevington , and F. C. Thompson . 2013. “Key to the Genera of Nearctic Syrphidae.” Canadian Journal of Arthropod Identification 23: 1–351. 10.3752/cjai.2013.23.

[ece372145-bib-0071] Montiel‐León, J. M. , G. Munoz , S. Vo Duy , et al. 2019. “Widespread Occurrence and Spatial Distribution of Glyphosate, Atrazine, and Neonicotinoids Pesticides in the St. Lawrence and Tributary Rivers.” Environmental Pollution 250: 29–39. 10.1016/j.envpol.2019.03.125. 30981933

[ece372145-bib-0072] Moquet, L. , E. Laurent , R. Bacchetta , and A. Jacquemart . 2018. “Conservation of Hoverflies (Diptera, Syrphidae) Requires Complementary Resources at the Landscape and Local Scales.” Insect Conservation and Diversity 11, no. 1: 72–87. 10.1111/icad.12245. 32336985 PMC7165621

[ece372145-bib-0073] Morandin, L. A. , R. F. Long , and C. Kremen . 2014. “Hedgerows Enhance Beneficial Insects on Adjacent Tomato Fields in an Intensive Agricultural Landscape.” Agriculture, Ecosystems & Environment 189: 164–170. 10.1016/j.agee.2014.03.030.

[ece372145-bib-0074] Moretti, M. , A. T. C. Dias , F. De Bello , et al. 2017. “Handbook of Protocols for Standardized Measurement of Terrestrial Invertebrate Functional Traits.” Functional Ecology 31, no. 3: 558–567. 10.1111/1365-2435.12776.

[ece372145-bib-0075] Moritz, C. , and C. Cicero . 2004. “DNA Barcoding: Promise and Pitfalls.” PLoS Biology 2, no. 10: e354. 10.1371/journal.pbio.0020354. 15486587 PMC519004

[ece372145-bib-0076] Musa, F. , D. Krasniqi , and S. Musa . 2020. “Aphid Complex Associated With Potato in Agro‐Climatic Conditions of Kosovo.” Agronomy Research 18: 206–215. 10.15159/AR.20.074.

[ece372145-bib-0077] Niemann, J.‐U. , and H.‐M. Poehling . 2022. “Effect of Narrow‐Banded Blue LED Device on Host Plant Settlement by Greenhouse Whitefly and Currant‐Lettuce Aphid.” Journal of Plant Diseases and Protection 129, no. 5: 1217–1225. 10.1007/s41348-022-00622-7.

[ece372145-bib-0078] Oksanen, J. , F. G. Blanchet , M. Friendly , et al. 2024. “vegan: Community Ecology Package (Version 2.7‐5) [R Package].” Comprehensive R Archive Network (CRAN). https://CRAN.R‐project.org/package=vegan.

[ece372145-bib-0079] Pascual‐Villalobos, M. J. , A. Lacasa , A. González , P. Varó , and M. J. García . 2006. “Effect of Flowering Plant Strips on Aphid and Syrphid Populations in Lettuce.” European Journal of Agronomy 24, no. 2: 182–185. 10.1016/j.eja.2005.07.003.

[ece372145-bib-0080] Picó, F. X. , and J. Retana . 2003. “Seed Ecology of a Mediterranean Perennial Herb With an Exceptionally Extended Flowering and Fruiting Season.” Botanical Journal of the Linnean Society 142, no. 3: 273–280. 10.1046/j.1095-8339.2003.00172.x.

[ece372145-bib-0081] Pielou, E. C. 1966. “The Measurement of Diversity in Different Types of Biological Collections.” Journal of Theoretical Biology 13: 131–144. 10.1016/0022-5193(66)90013-0.

[ece372145-bib-0082] Popović, D. , J. Ačanski , M. Djan , D. Obreht , A. Vujić , and S. Radenković . 2015. “Sibling Species Delimitation and Nomenclature of the *Merodon avidus* Complex (Diptera: Syrphidae).” European Journal of Entomology 112, no. 4: 790–809. 10.14411/eje.2015.100.

[ece372145-bib-0083] Puillandre, N. , S. Brouillet , and G. Achaz . 2021. “ASAP: Assemble Species by Automatic Partitioning.” Molecular Ecology Resources 21, no. 2: 609–620. 10.1111/1755-0998.13281. 33058550

[ece372145-bib-0084] R Development Core Team . 2024. R: A Language and Environment for Statistical Computing (Version 4.5.0) [Computer Software]. R Foundation for Statistical Computing. https://www.R‐project.org/.

[ece372145-bib-0085] Ratnasingham, S. , and P. D. N. Hebert . 2013. “A DNA‐Based Registry for All Animal Species: The Barcode Index Number (BIN) System.” PLoS One 8, no. 7: e66213. 10.1371/journal.pone.0066213. 23861743 PMC3704603

[ece372145-bib-0086] Raymond, L. , M. Plantegenest , and A. Vialatte . 2013. “Migration and Dispersal May Drive to High Genetic Variation and Significant Genetic Mixing: The Case of Two Agriculturally Important, Continental Hoverflies (* episyrphus balteatus and Sphaerophoria scripta*).” Molecular Ecology 22, no. 21: 5329–5339. 10.1111/mec.12483. 24138027

[ece372145-bib-0087] Ribeiro, A. L. , and L. M. Gontijo . 2017. “Alyssum Flowers Promote Biological Control of Collard Pests.” Biocontrol 62, no. 2: 185–196. 10.1007/s10526-016-9783-7.

[ece372145-bib-0088] Rodríguez‐Gasol, N. , G. Alins , E. R. Veronesi , and S. Wratten . 2020. “The Ecology of Predatory Hoverflies as Ecosystem‐Service Providers in Agricultural Systems.” Biological Control 151: 104405. 10.1016/j.biocontrol.2020.104405.

[ece372145-bib-0089] Rufingier, C. , L. Schoen , C. Martin , and N. Pasteur . 1997. “Resistance of *Nasonovia ribisnigri* (Homoptera: Aphididae) to Five Insecticides.” Journal of Economic Entomology 90, no. 6: 1445–1449. 10.1093/jee/90.6.1445.

[ece372145-bib-0091] Scarlato, M. , L. Bao , W. A. H. Rossing , S. Dogliotti , P. Bertoni , and F. J. J. A. Bianchi . 2023. “Flowering Plants in Open Tomato Greenhouses Enhance Pest Suppression in Conventional Systems and Reveal Resource Saturation for Natural Enemies in Organic Systems.” Agriculture, Ecosystems & Environment 347: 108389. 10.1016/j.agee.2023.108389.

[ece372145-bib-0092] Scheper, J. , A. Holzschuh , M. Kuussaari , et al. 2015. “Local and Landscape‐Level Floral Resources Explain Effects of Wildflower Strips on Wild Bees Across Four European Countries.” Journal of Applied Ecology 52, no. 5: 1165–1175. 10.1111/1365-2664.12479.

[ece372145-bib-0093] Shannon, C. E. 1948. “A Mathematical Theory of Communication.” Bell System Technical Journal 27, no. 3: 379–423. 10.1002/j.1538-7305.1948.tb01338.x.

[ece372145-bib-0094] Shapiro, S. S. , and M. B. Wilk . 1965. “An Analysis of Variance Test for Normality (Complete Samples).” Biometrika 52, no. 3–4: 591–611. 10.1093/biomet/52.3-4.591.

[ece372145-bib-0095] Simpson, E. H. 1949. “Measurement of Diversity.” Nature 163, no. 4148: 688. 10.1038/163688a0.

[ece372145-bib-0096] Skevington, J. , M. Locke , A. Young , K. Moran , W. Crins , and S. Marshall . 2019. Field Guide to the Flower Flies of Northeastern North America. Princeton University Press. 10.1515/9780691192512.

[ece372145-bib-0097] Smith, H. A. , and W. E. Chaney . 2007. “A Survey of Syrphid Predators of *Nasonovia ribisnigri* in Organic Lettuce on the Central Coast of California.” Journal of Economic Entomology 100, no. 1: 39–48. 10.1603/0022-0493(2007)100[39:ASOSPO]2.0.CO;2. 17370807

[ece372145-bib-0098] Smith, H. A. , W. E. Chaney , and T. A. Bensen . 2008. “Role of Syrphid Larvae and Other Predators in Suppressing Aphid Infestations in Organic Lettuce on California's Central Coast.” Journal of Economic Entomology 101, no. 5: 1526–1532. 10.1603/0022-0493(2008)101[1526:ROSLAO]2.0.CO;2. 18950033

[ece372145-bib-0099] Star, B. , and H. G. Spencer . 2013. “Effects of Genetic Drift and Gene Flow on the Selective Maintenance of Genetic Variation.” Genetics 194, no. 1: 235–244. 10.1534/genetics.113.149781. 23457235 PMC3632471

[ece372145-bib-0100] Stufkens, M. A. W. , and D. A. J. Teulon . 2003. “Distribution Host Range and Flight Pattern of the Lettuce Aphid in New Zealand.” New Zealand Plant Protection 56: 27–32. 10.30843/nzpp.2003.56.6027.

[ece372145-bib-0101] Tschumi, M. , M. Albrecht , C. Bärtschi , J. Collatz , M. H. Entling , and K. Jacot . 2016. “Perennial, Species‐Rich Wildflower Strips Enhance Pest Control and Crop Yield.” Agriculture, Ecosystems & Environment 220: 97–103. 10.1016/j.agee.2016.01.001.

[ece372145-bib-0102] Tschumi, M. , M. Albrecht , J. Collatz , et al. 2016. “Tailored Flower Strips Promote Natural Enemy Biodiversity and Pest Control in Potato Crops.” Journal of Applied Ecology 53, no. 4: 1169–1176. 10.1111/1365-2664.12653.

[ece372145-bib-0103] Van Veen, M. P. 2010. “Hoverflies of Northwest Europe: Identification Keys to the Syrphidae.” 10.13140/RG.2.1.3697.4567.

[ece372145-bib-0104] Violle, C. , M. Navas , D. Vile , et al. 2007. “Let the Concept of Trait Be Functional!” Oikos 116, no. 5: 882–892. 10.1111/j.0030-1299.2007.15559.x.

[ece372145-bib-0105] Virgilio, M. , T. Backeljau , B. Nevado , and M. De Meyer . 2010. “Comparative Performances of DNA Barcoding Across Insect Orders.” BMC Bioinformatics 11, no. 1: 206. 10.1186/1471-2105-11-206. 20420717 PMC2885370

[ece372145-bib-0106] Wong, M. K. L. , B. Guénard , and O. T. Lewis . 2019. “Trait‐Based Ecology of Terrestrial Arthropods.” Biological Reviews 94, no. 3: 999–1022. 10.1111/brv.12488. 30548743 PMC6849530

[ece372145-bib-0107] Wratten, S. D. , M. H. Bowie , J. M. Hickman , A. M. Evans , J. R. Sedcole , and J. M. Tylianakis . 2003. “Field Boundaries as Barriers to Movement of Hover Flies (Diptera: Syrphidae) in Cultivated Land.” Oecologia 134, no. 4: 605–611. 10.1007/s00442-002-1128-9. 12647134

[ece372145-bib-0108] Young, A. D. , A. R. Lemmon , J. H. Skevington , et al. 2016. “Anchored Enrichment Dataset for True Flies (Order Diptera) Reveals Insights Into the Phylogeny of Flower Flies (Family Syrphidae).” BMC Evolutionary Biology 16, no. 1: 143. 10.1186/s12862-016-0714-0. 27357120 PMC4928351

[ece372145-bib-0109] Zhang, H. , and W. Bu . 2022. “Exploring Large‐Scale Patterns of Genetic Variation in the COI Gene Among Insecta: Implications for DNA Barcoding and Threshold‐Based Species Delimitation Studies.” Insects 13, no. 5: 425. 10.3390/insects13050425. 35621761 PMC9147995

[ece372145-bib-0110] Zhong, J. , W. Pan , S. Jiang , et al. 2024. “Evaluating the Impact of Alyssum Flower Strips on Biological Control of Key Pests in Flue‐Cured Tobacco Agroecosystems.” Journal of Applied Entomology 148: jen.13337. 10.1111/jen.13337.

