# Supplementary material for: Syrphid Diversity in Sweet Alyssum Flower Strips in Quebec's Lettuce Fields: Molecular Identification and Delimitation of the Sphaerophoria Complex
Source: Ecol Evol. 2025 Sep 11;15(9):e72145. doi: 10.1002/ece3.72145 (PMC12425499; doi:10.1002/ece3.72145)
Supplement: Supplementary file 1 — Data S1: ece372145‐sup‐0001‐Supinfo.docx. [file ECE3-15-e72145-s001.docx]

**Supplementary materials**


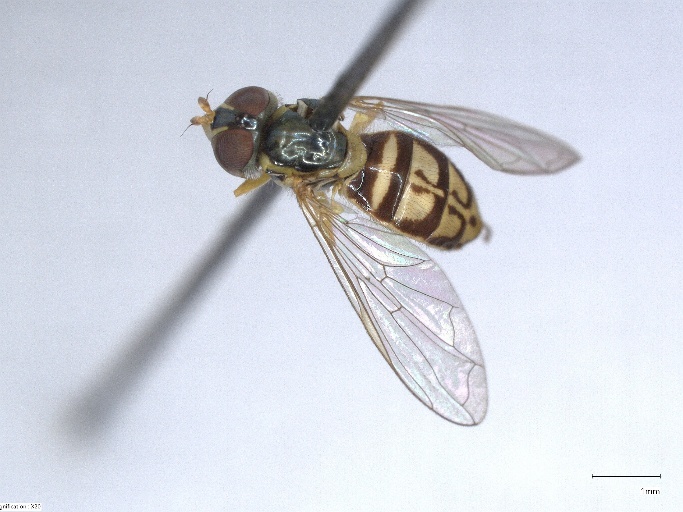


**Figure S1.** Female adult of the most abundant species, *Toxomerus marginatus* (Say) (QMOR93537).


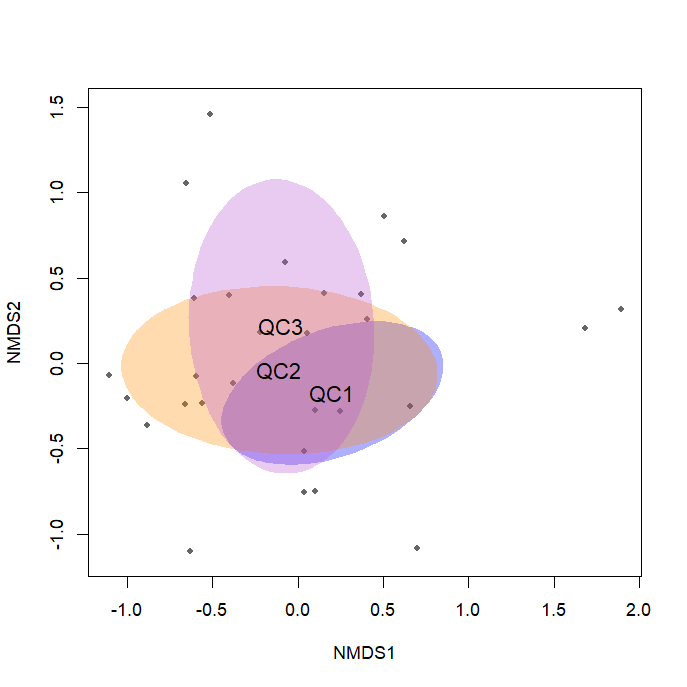


**Figure S2.** Non-metric multidimensional scaling (NMDS) plot of Syrphidae species collected in three different sites in Quebec, Canada. Each point represents a sampling date.

**Table S1. Summary of sampling details by site**

| Site | GPS coordinate | Sampling date | Sampling number | Syrphid total abundance |
| --- | --- | --- | --- | --- |
| QC1 | 45.193417, -73.344583 | 7/18/2022 | 1 | 10 |
|  |  | 7/22/2022 | 2 | 8 |
|  |  | 7/25/2022 | 3 | 8 |
|  |  | 8/1/2022 | 4 | 151 |
|  |  | 8/5/2022 | 5 | 172 |
|  |  | 8/15/2022 | 6 | 177 |
|  |  | 8/19/2022 | 7 | 366 |
|  |  | 8/22/2022 | 8 | 197 |
| QC2 | 45.148107, -73.494923 | 7/11/2022 | 1 | 3 |
|  |  | 7/15/2022 | 2 | 5 |
|  |  | 7/18/2022 | 3 | 27 |
|  |  | 7/22/2022 | 4 | 24 |
|  |  | 7/25/2022 | 5 | 33 |
|  |  | 7/29/2022 | 6 | 10 |
|  |  | 8/2/2022 | 7 | 94 |
|  |  | 8/5/2022 | 8 | 208 |
| QC3 | 45.143579, -73.438052 | 7/18/2022 | 1 | 3 |
|  |  | 7/25/2022 | 2 | 1 |
|  |  | 8/2/2022 | 3 | 61 |
|  |  | 8/5/2022 | 4 | 65 |
|  |  | 8/12/2022 | 5 | 174 |
|  |  | 8/15/2022 | 6 | 2 |
|  |  | 8/22/2022 | 7 | 31 |
|  |  | 8/28/2022 | 8 | 102 |

**Table S2.** DNA barcoding success rates for syrphid species. Number of specimens processed and successfully barcoded

| **Syrphid species** | **Specimens subjected to DNA barcoding** | **Successfully barcoded specimens** | **Barcode Index Number BIN** |
| --- | --- | --- | --- |
| *Allograpta obliqua* | 4 | 4 | [BOLD:AAD8276](https://v4.boldsystems.org/index.php/Public_BarcodeCluster?clusteruri=BOLD:AAD8276) |
| *Eristalis arbustorum* | 4 | 4 | [BOLD:AAA8223](https://v4.boldsystems.org/index.php/Public_BarcodeCluster?clusteruri=BOLD:AAA8223) |
| *Eupeodes americanus* | 17 | 16 | [BOLD:AAB2384](https://v4.boldsystems.org/index.php/Public_BarcodeCluster?clusteruri=BOLD:AAB2384) |
| *Melanostoma mellinum* | 2 | 2 | [BOLD:AAB2866](https://v4.boldsystems.org/index.php/Public_BarcodeCluster?clusteruri=BOLD:AAB2866) |
| *Platycheirus quadratus* | 2 | 2 | [BOLD:AAA9506](https://v4.boldsystems.org/index.php/Public_BarcodeCluster?clusteruri=BOLD:AAA9506) |
| *Syrphus rectus* | 10 | 10 | [BOLD:AAB5577](https://v4.boldsystems.org/index.php/Public_BarcodeCluster?clusteruri=BOLD:AAB5577) |
| *Syrphus ribesii* | 2 | 2 | [BOLD:AAA4570](https://v4.boldsystems.org/index.php/Public_BarcodeCluster?clusteruri=BOLD:AAA4570) |
| *Syrphus knabi* | 4 | 4 | [BOLD:AAG4667](https://v4.boldsystems.org/index.php/Public_BarcodeCluster?clusteruri=BOLD:AAG4667) |
| *Toxomerus marginatus* | 3 | 2 | [BOLD:AAA4277](https://v4.boldsystems.org/index.php/Public_BarcodeCluster?clusteruri=BOLD:AAA4277) |
| *Toxomerus geminatus* | 3 | 3 | [BOLD:AAC1312](https://v4.boldsystems.org/index.php/Public_BarcodeCluster?clusteruri=BOLD:AAC1312) |
| *Sphaerophoria philanthus* | 41 | 41 | [BOLD:AAA7374](https://v4.boldsystems.org/index.php/Public_BarcodeCluster?clusteruri=BOLD:AAA7374) |
| *Sphaerophoria contigua* | 7 | 5 | [BOLD:AAA7374](https://v4.boldsystems.org/index.php/Public_BarcodeCluster?clusteruri=BOLD:AAA7374) |
| *Sphaerophoria* complex* | 82 | 41 | [BOLD:AAA7374](https://v4.boldsystems.org/index.php/Public_BarcodeCluster?clusteruri=BOLD:AAA7374) |
| Total | 181 | 134 |  |

* *Sphaerophoria* complex = *philanthus/asymmetrica/abbreviata*

**Table S3:** *Sphaerophoria* haplotypes and MOTU delimitated by ASAP

| Sequence ID | Haplotypes | ASAP1 | ASAP2 |
| --- | --- | --- | --- |
| *LRSYR030-07\|Sphaerophoria philanthus\|COI-5P\|KR654858* | H1 | MOTU1 | MOTU1 |
| *LRSYR045-07\|Sphaerophoria philanthus\|COI-5P\|KR660492* | H1 | MOTU1 | MOTU1 |
| *BCBEE552-22\|Sphaerophoria philanthus\|COI-5P* | H1 | MOTU1 | MOTU1 |
| *MHSYR034-06\|Sphaerophoria philanthus\|COI-5P\|PP418754* | H1 | MOTU1 | MOTU1 |
| *SSBAA5584-12\|Sphaerophoria scripta\|COI-5P\|KM928358* | H1 | MOTU1 | MOTU1 |
| *JWDCJ1406-11\|Sphaerophoria philanthus\|COI-5P\|JN285900* | H1 | MOTU1 | MOTU1 |
| *NBPP257-18\|Sphaerophoria scripta\|COI-5P\|OQ622870* | H1 | MOTU1 | MOTU1 |
| *SSBAA5583-12\|Sphaerophoria scripta\|COI-5P\|KM928507* | H1 | MOTU1 | MOTU1 |
| *MHSYR059-07\|Sphaerophoria philanthus\|COI-5P\|PP418226* | H1 | MOTU1 | MOTU1 |
| *JSSEP855-11\|Sphaerophoria scripta\|COI-5P\|KR658034* | H1 | MOTU1 | MOTU1 |
| *LRSYR004-07\|Sphaerophoria philanthus\|COI-5P\|KR671982* | H1 | MOTU1 | MOTU1 |
| *CNGBJ1911-14\|Sphaerophoria scripta\|COI-5P\|KR393451* | H1 | MOTU1 | MOTU1 |
| *SSBAD5318-13\|Sphaerophoria scripta\|COI-5P\|KM934590* | H1 | MOTU1 | MOTU1 |
| *TTDFW249-08\|Sphaerophoria scripta\|COI-5P\|KM570562* | H1 | MOTU1 | MOTU1 |
| *TTDFW805-08\|Sphaerophoria scripta\|COI-5P\|KM570700* | H1 | MOTU1 | MOTU1 |
| *CNJAG1182-12\|Sphaerophoria scripta\|COI-5P\|KR465851* | H1 | MOTU1 | MOTU1 |
| *JWDCJ1404-11\|Sphaerophoria philanthus\|COI-5P\|JN285899* | H1 | MOTU1 | MOTU1 |
| *BBDEC354-09\|Sphaerophoria scripta\|COI-5P\|HM412046* | H1 | MOTU1 | MOTU1 |
| *NBPP288-18\|Sphaerophoria scripta\|COI-5P\|OQ622864* | H1 | MOTU1 | MOTU1 |
| *TTDFW745-08\|Sphaerophoria scripta\|COI-5P\|KM569911* | H1 | MOTU1 | MOTU1 |
| *TTDFW903-08\|Sphaerophoria contigua\|COI-5P\|KM569727* | H1 | MOTU1 | MOTU1 |
| *CNCDB3248-11\|Sphaerophoria philanthus\|COI-5P* | H1 | MOTU1 | MOTU1 |
| *JSFLA011-08\|Sphaerophoria philanthus\|COI-5P\|KR663928* | H1 | MOTU1 | MOTU1 |
| *JSYKA277-10\|Sphaerophoria scripta\|COI-5P\|HM860420* | H1 | MOTU1 | MOTU1 |
| *TTDFW798-08\|Sphaerophoria scripta\|COI-5P\|KM570358* | H1 | MOTU1 | MOTU1 |
| *TTDFW806-08\|Sphaerophoria scripta\|COI-5P\|KM569848* | H1 | MOTU1 | MOTU1 |
| *CNCDB3245-11\|Sphaerophoria philanthus\|COI-5P\|KC900490* | H1 | MOTU1 | MOTU1 |
| *NBPP225-18\|Sphaerophoria scripta\|COI-5P\|OQ622854* | H1 | MOTU1 | MOTU1 |
| *CNCDB3215-11\|Sphaerophoria cleoae\|COI-5P* | H1 | MOTU1 | MOTU1 |
| *CNCDB3217-11\|Sphaerophoria contigua\|COI-5P* | H1 | MOTU1 | MOTU1 |
| *CNCDB3219-11\|Sphaerophoria contigua\|COI-5P* | H1 | MOTU1 | MOTU1 |
| *JWDCL407-11\|Sphaerophoria philanthus\|COI-5P\|KR653967* | H1 | MOTU1 | MOTU1 |
| *TTDFW794-08\|Sphaerophoria scripta\|COI-5P\|KM570778* | H1 | MOTU1 | MOTU1 |
| *CNCDB2235-11\|Sphaerophoria asymmetrica\|COI-5P* | H1 | MOTU1 | MOTU1 |
| *CNCDB3219-11\|Sphaerophoria contigua\|COI-5P* | H1 | MOTU1 | MOTU1 |
| *CNCDB2318-11\|Sphaerophoria philanthus\|COI-5P* | H1 | MOTU1 | MOTU1 |
| *CNCDB3196-11\|Sphaerophoria abbreviata\|COI-5P* | H1 | MOTU1 | MOTU1 |
| *BBDCM674-10\|Sphaerophoria scripta\|COI-5P\|JF867368* | H1 | MOTU1 | MOTU1 |
| *JWDCJ850-11\|Sphaerophoria philanthus\|COI-5P\|KR673094* | H1 | MOTU1 | MOTU1 |
| *JWDCK239-11\|Sphaerophoria philanthus\|COI-5P\|JF878166* | H1 | MOTU1 | MOTU1 |
| *JSDIQ785-10\|Sphaerophoria scripta\|COI-5P\|JF873776* | H1 | MOTU1 | MOTU1 |
| *JWDCE919-10\|Sphaerophoria philanthus\|COI-5P\|JF874863* | H1 | MOTU1 | MOTU1 |
| *JWDCK236-11\|Sphaerophoria philanthus\|COI-5P\|JF878163* | H1 | MOTU1 | MOTU1 |
| *NBPP381-18\|Sphaerophoria scripta\|COI-5P\|OQ622860* | H1 | MOTU1 | MOTU1 |
| *CNCDB3221-11\|Sphaerophoria contigua\|COI-5P* | H1 | MOTU1 | MOTU1 |
| *JWDCH819-10\|Sphaerophoria philanthus\|COI-5P\|JF877000* | H1 | MOTU1 | MOTU1 |
| *LYMAB308-15\|Sphaerophoria philanthus\|COI-5P* | H1 | MOTU1 | MOTU1 |
| *JWDCK240-11\|Sphaerophoria philanthus\|COI-5P\|JF878167* | H1 | MOTU1 | MOTU1 |
| *MHSYR095-07\|Sphaerophoria philanthus\|COI-5P\|PP418435* | H1 | MOTU1 | MOTU1 |
| *BBDCP939-10\|Sphaerophoria scripta\|COI-5P\|JF869292* | H1 | MOTU1 | MOTU1 |
| *CNCDB061-11\|Sphaerophoria asymmetrica\|COI-5P\|KC900483* | H1 | MOTU1 | MOTU1 |
| *JWDCK125-11\|Sphaerophoria philanthus\|COI-5P\|KR667239* | H1 | MOTU1 | MOTU1 |
| *MHSYR102-07\|Sphaerophoria philanthus\|COI-5P\|PP418663* | H1 | MOTU1 | MOTU1 |
| *NBPP013-18\|Sphaerophoria scripta\|COI-5P\|OQ622876* | H1 | MOTU1 | MOTU1 |
| *CNCDB3203-11\|Sphaerophoria asymmetrica\|COI-5P* | H1 | MOTU1 | MOTU1 |
| *CNCDB2236-11\|Sphaerophoria asymmetrica\|COI-5P* | H1 | MOTU1 | MOTU1 |
| *BBDCP933-10\|Sphaerophoria scripta\|COI-5P\|JF869286* | H1 | MOTU1 | MOTU1 |
| *CNCDB3220-11\|Sphaerophoria contigua\|COI-5P\|KC900440* | H1 | MOTU1 | MOTU1 |
| *MHSYR297-07\|Sphaerophoria philanthus\|COI-5P\|PP418351* | H1 | MOTU1 | MOTU1 |
| *NBPP252-18\|Sphaerophoria scripta\|COI-5P\|OQ622869* | H1 | MOTU1 | MOTU1 |
| *NBPP509-18\|Sphaerophoria scripta\|COI-5P\|OQ622857* | H1 | MOTU1 | MOTU1 |
| *CNCDB3249-11\|Sphaerophoria philanthus\|COI-5P* | H1 | MOTU1 | MOTU1 |
| *JWDCG052-10\|Sphaerophoria philanthus\|COI-5P\|JF875749* | H1 | MOTU1 | MOTU1 |
| *JWDCG053-10\|Sphaerophoria philanthus\|COI-5P\|JF875750* | H1 | MOTU1 | MOTU1 |
| *BCBEE948-22\|Sphaerophoria brevipilosa\|COI-5P* | H1 | MOTU1 | MOTU1 |
| *MHSYR108-07\|Sphaerophoria philanthus\|COI-5P\|PP418206* | H1 | MOTU1 | MOTU1 |
| *LYMAB311-15\|Sphaerophoria philanthus\|COI-5P* | H1 | MOTU1 | MOTU1 |
| *E2-Sphaerophoria philanthus/asymmetrica/abbreviata* | H1 | MOTU1 | MOTU1 |
| *E3-Sphaerophoria philanthus/asymmetrica/abbreviata* | H1 | MOTU1 | MOTU1 |
| *E12-Sphaerophoria philanthus/asymmetrica/abbreviata* | H1 | MOTU1 | MOTU1 |
| *E17-Sphaerophoria philanthus* | H1 | MOTU1 | MOTU1 |
| *E24-Sphaerophoria philanthus* | H1 | MOTU1 | MOTU1 |
| *E27-Sphaerophoria philanthus/asymmetrica/abbreviata* | H1 | MOTU1 | MOTU1 |
| *E35-Sphaerophoria philanthus* | H1 | MOTU1 | MOTU1 |
| *E36-Sphaerophoria philanthus* | H1 | MOTU1 | MOTU1 |
| *E41-Sphaerophoria philanthus/asymmetrica/abbreviata* | H1 | MOTU1 | MOTU1 |
| *E43-Sphaerophoria philanthus* | H1 | MOTU1 | MOTU1 |
| *E52-Sphaerophoria philanthus* | H1 | MOTU1 | MOTU1 |
| *E56-Sphaerophoria philanthus* | H1 | MOTU1 | MOTU1 |
| *E72-Sphaerophoria philanthus/asymmetrica/abbreviata* | H1 | MOTU1 | MOTU1 |
| *E75-Sphaerophoria philanthus* | H1 | MOTU1 | MOTU1 |
| *E78-Sphaerophoria philanthus* | H1 | MOTU1 | MOTU1 |
| *E81-Sphaerophoria philanthus* | H1 | MOTU1 | MOTU1 |
| *E86-Sphaerophoria philanthus/asymmetrica/abbreviata* | H1 | MOTU1 | MOTU1 |
| *E94-Sphaerophoria philanthus* | H1 | MOTU1 | MOTU1 |
| *E96-Sphaerophoria philanthus* | H1 | MOTU1 | MOTU1 |
| *E102-Sphaerophoria philanthus/asymmetrica/abbreviata* | H1 | MOTU1 | MOTU1 |
| *E112-Sphaerophoria philanthus/asymmetrica/abbreviata* | H1 | MOTU1 | MOTU1 |
| *E117-Sphaerophoria philanthus/asymmetrica/abbreviata* | H1 | MOTU1 | MOTU1 |
| *E118-Sphaerophoria philanthus* | H1 | MOTU1 | MOTU1 |
| *E120-Sphaerophoria philanthus/asymmetrica/abbreviata* | H1 | MOTU1 | MOTU1 |
| *E124-Sphaerophoria philanthus/asymmetrica/abbreviata* | H1 | MOTU1 | MOTU1 |
| *JWDCI418-10\|Sphaerophoria philanthus\|COI-5P\|KR662069* | H1 | MOTU1 | MOTU1 |
| *MHSYR095-07\|Sphaerophoria philanthus\|COI-5P\|PP418435* | H1 | MOTU1 | MOTU1 |
| *INRMA4367-21\|Sphaerophoria\|COI-5P* | H1 | MOTU1 | MOTU1 |
| *AGAKQ2797-17\|Sphaerophoria\|COI-5P\|MG168892* | H1 | MOTU1 | MOTU1 |
| *AGAKM152-17\|Sphaerophoria\|COI-5P\|MG169378* | H1 | MOTU1 | MOTU1 |
| *CNSIE1394-15\|Sphaerophoria\|COI-5P\|MF830935* | H1 | MOTU1 | MOTU1 |
| *CNSIE1396-15\|Sphaerophoria\|COI-5P\|MF838071* | H1 | MOTU1 | MOTU1 |
| *ABOTH3619-22\|Sphaerophoria\|COI-5P* | H1 | MOTU1 | MOTU1 |
| *TTDFW658-08\|Sphaerophoria\|COI-5P\|KM570801* | H1 | MOTU1 | MOTU1 |
| *SMTPI7717-14\|Sphaerophoria\|COI-5P\|KR763557* | H1 | MOTU1 | MOTU1 |
| *AGAKQ256-17\|Sphaerophoria\|COI-5P\|MG164443* | H1 | MOTU1 | MOTU1 |
| *SSROC035-14\|Sphaerophoria\|COI-5P\|KR525502* | H1 | MOTU1 | MOTU1 |
| *CNCDB3197-11\|Sphaerophoria abbreviata\|COI-5P* | H10 | MOTU1 | MOTU1 |
| *CNCDB057-11\|Sphaerophoria abbreviata\|COI-5P* | H11 | MOTU1 | MOTU1 |
| *E105-Sphaerophoria philanthus/asymmetrica/abbreviata* | H12 | MOTU1 | MOTU1 |
| *OPPEC4918-17\|Sphaerophoria philanthus\|COI-5P* | H13 | MOTU1 | MOTU1 |
| *JWDCL247-11\|Sphaerophoria philanthus\|COI-5P\|KR659153* | H14 | MOTU1 | MOTU1 |
| *MHSYR296-07\|Sphaerophoria philanthus\|COI-5P\|PP418695* | H14 | MOTU1 | MOTU1 |
| *E23-sphaerophoria philanthus/asymmetrica/abbreviata* | H15 | MOTU1 | MOTU1 |
| *E33-Sphaerophoria philanthus/asymmetrica/abbreviata* | H15 | MOTU1 | MOTU1 |
| *E51-Sphaerophoria philanthus* | H16 | MOTU1 | MOTU1 |
| *E70-Sphaerophoria philanthus* | H16 | MOTU1 | MOTU1 |
| *NBPP218-18\|Sphaerophoria scripta\|COI-5P\|OQ622881* | H17 | MOTU1 | MOTU1 |
| *E32-Sphaerophoria philanthus/asymmetrica/abbreviata* | H18 | MOTU1 | MOTU1 |
| *E25-Sphaerophoria philanthus/asymmetrica/abbreviata* | H19 | MOTU1 | MOTU1 |
| *CNCDB128-11\|Sphaerophoria contigua\|COI-5P* | H2 | MOTU1 | MOTU1 |
| *LYMAD1107-19\|Sphaerophoria contigua\|COI-5P* | H2 | MOTU1 | MOTU1 |
| *CNCDB3917-11\|Sphaerophoria contigua\|COI-5P* | H2 | MOTU1 | MOTU1 |
| *E113-Sphaerophoria philanthus/asymmetrica/abbreviata* | H2 | MOTU1 | MOTU1 |
| *E114-Sphaerophoria philanthus/asymmetrica/abbreviata* | H2 | MOTU1 | MOTU1 |
| *E119-Sphaerophoria philanthus/asymmetrica/abbreviata* | H2 | MOTU1 | MOTU1 |
| *E125-Sphaerophoria philanthus/asymmetrica/abbreviata* | H2 | MOTU1 | MOTU1 |
| *E57-Sphaerophoria philanthus/asymmetrica/abbreviata* | H20 | MOTU1 | MOTU1 |
| *CANIN334-18\|Sphaerophoria philanthus\|COI-5P* | H21 | MOTU1 | MOTU1 |
| *E115-Sphaerophoria philanthus/asymmetrica/abbreviata* | H22 | MOTU1 | MOTU1 |
| *E109-Sphaerophoria philanthus/asymmetrica/abbreviata* | H23 | MOTU1 | MOTU1 |
| *E104-Sphaerophoria philanthus* | H24 | MOTU1 | MOTU1 |
| *E121-Sphaerophoria philanthus* | H24 | MOTU1 | MOTU1 |
| *E19-Sphaerophoria philanthus/asymmetrica/abbreviata* | H25 | MOTU1 | MOTU1 |
| *E58-Sphaerophoria philanthus/asymmetrica/abbreviata* | H26 | MOTU1 | MOTU1 |
| *E60-Sphaerophoria philanthus/asymmetrica/abbreviata* | H26 | MOTU1 | MOTU1 |
| *E4-Sphaerohoria philanthus/asymmetrica/abbreviata* | H27 | MOTU1 | MOTU1 |
| *E66-Sphaerophoria philanthus/asymmetrica/abbreviata* | H27 | MOTU1 | MOTU1 |
| *E34-Sphaerophoria philanthus/asymmetrica/abbreviata* | H28 | MOTU2 | MOTU2 |
| *E97-Sphaerophoria philanthus/asymmetrica/abbreviata* | H28 | MOTU2 | MOTU2 |
| *JWDCE918-10\|Sphaerophoria philanthus\|COI-5P\|JF874862* | H3 | MOTU1 | MOTU1 |
| *JWDCH675-10\|Sphaerophoria philanthus\|COI-5P\|JF876894* | H3 | MOTU1 | MOTU1 |
| *LRSYR035-07\|Sphaerophoria philanthus\|COI-5P\|KR653748* | H3 | MOTU1 | MOTU1 |
| *JWDCJ733-11\|Sphaerophoria philanthus\|COI-5P\|KR662664* | H3 | MOTU1 | MOTU1 |
| *CNCDB3201-11\|Sphaerophoria asymmetrica\|COI-5P* | H3 | MOTU1 | MOTU1 |
| *LRSYR038-07\|Sphaerophoria philanthus\|COI-5P\|KR671288* | H3 | MOTU1 | MOTU1 |
| *JWDCE918-10\|Sphaerophoria_2 philanthus\|COI-5P\|JF874862* | H3 | MOTU1 | MOTU1 |
| *E84-Sphaerophoria philanthus/asymmetrica/abbreviata* | H31 | MOTU1 | MOTU1 |
| *E85-Sphaerophoria philanthus/asymmetrica/abbreviata* | H32 | MOTU1 | MOTU1 |
| *E39-Sphaerophoria philanthus/asymmetrica/abbreviata* | H33 | MOTU1 | MOTU1 |
| *E71-Sphaerophoria philanthus/asymmetrica/abbreviata* | H34 | MOTU1 | MOTU1 |
| *E76-Sphaerophoria philanthus/asymmetrica/abbreviata* | H35 | MOTU1 | MOTU1 |
| *E10-Sphaerophoria philanthus/asymmetrica/abbreviata* | H36 | MOTU1 | MOTU1 |
| *E65-Sphaerophoria philanthus/asymmetrica/abbreviata* | H37 | MOTU1 | MOTU1 |
| *E47-Sphaerophoria philanthus* | H38 | MOTU1 | MOTU1 |
| *CNCDB3205-11\|Sphaerophoria asymmetrica\|COI-5P* | H39 | MOTU1 | MOTU1 |
| *CNCDB3228-11\|Sphaerophoria longipilosa\|COI-5P* | H4 | MOTU1 | MOTU1 |
| *CNCDB3229-11\|Sphaerophoria longipilosa\|COI-5P* | H4 | MOTU1 | MOTU1 |
| *CNCDB3231-11\|Sphaerophoria longipilosa\|COI-5P* | H4 | MOTU1 | MOTU1 |
| *CNCDB2320-11\|Sphaerophoria longipilosa\|COI-5P* | H4 | MOTU1 | MOTU1 |
| *CNCDB3232-11\|Sphaerophoria longipilosa\|COI-5P* | H4 | MOTU1 | MOTU1 |
| *CNCDB062-11\|Sphaerophoria bifurcata\|COI-5P* | H5 | MOTU1 | MOTU1 |
| *CNCDB2321-11\|Sphaerophoria bifurcata\|COI-5P* | H5 | MOTU1 | MOTU1 |
| *CNCDB058-11\|Sphaerophoria abbreviata\|COI-5P* | H5 | MOTU1 | MOTU1 |
| *LYMAD1104-19\|Sphaerophoria cranbrookensis\|COI-5P* | H5 | MOTU1 | MOTU1 |
| *CNCDB2322-11\|Sphaerophoria bifurcata\|COI-5P* | H5 | MOTU1 | MOTU1 |
| *CNCDB2233-11\|Sphaerophoria abbreviata\|COI-5P* | H5 | MOTU1 | MOTU1 |
| *E1-Sphaerophoria philanthus/asymmetrica/abbreviata* | H6 | MOTU1 | MOTU1 |
| *E5-Sphaerophoria philanthus* | H6 | MOTU1 | MOTU1 |
| *E30-Sphaerophoria philanthus* | H6 | MOTU1 | MOTU1 |
| *E45-Sphaerophoriaphilanthus* | H6 | MOTU1 | MOTU1 |
| *NBPP129-18\|Sphaerophoria scripta\|COI-5P\|OQ622862* | H7 | MOTU1 | MOTU1 |
| *E110-Sphaerophoria philanthus/asymmetrica/abbreviata* | H7 | MOTU1 | MOTU1 |
| *NBPP289-18\|Sphaerophoria scripta\|COI-5P\|OQ622865* | H8 | MOTU1 | MOTU1 |
| *CNCDB3199-11\|Sphaerophoria abbreviata\|COI-5P* | H9 | MOTU1 | MOTU1 |
| *E44-Sphaerophoria philanthus/asymmetrica/abbreviata* | H29 | MOTU1 | MOTU3 |
| *E8-Sphaerophoria philanthus/asymmetrica/abbreviata* | H30 | MOTU1 | MOTU3 |

**Table S4:** The ten most-supported partitions generated by Assemble Species by Automatic Partitioning (ASAP). The partitions marked with an asterisk represent the clustering levels that the algorithm considers most relevant or optimal for describing the data structure.

| Number of subsets | Asap-score | P-val (rank) | W (rank) | Treshold distance |
| --- | --- | --- | --- | --- |
| * 2 | 1.50 | 5.49e-01 (2) | 3.76e-06 (1) | 0.013556 |
| * 3 | 4.50 | 7.66e-01 (5) | 2.38e-06 (4) | 0.010888 |
| 43 | 5.00 | 5.31e-01 (1) | 1.70e-06 (9) | 0.001532 |
| 46 | 8.00 | 8.72e-01 (8) | 1.70e-06 (8) | 0.001529 |
| 20 | 8.00 | 9.20e-01 (13) | 3.39e-06 (3) | 0.003587 |
| 45 | 8.50 | 8.94e-01 (10) | 1.70e-06 (7) | 0.001530 |
| * 11 | 11.50 | 9.30e-01 (17) | 1.89e-06 (6) | 0.005141 |
| 42 | 12.00 | 9.24e-01 (14) | 1.70e-06 (10) | 0.001539 |
| 48 | 13.50 | 9.24e-01 (15) | 1.69e-06 (12) | 0.001524 |
| 29 | 14.00 | 9.70e-01 (26) | 3.55e-06 (2) | 0.001734 |
